# Supplementary material for: Life habits and evolutionary biology of new two-winged long-proboscid scorpionflies from mid-Cretaceous Myanmar amber
Source: Nat Commun. 2019 Mar 15;10:1235. doi: 10.1038/s41467-019-09236-4 (PMC6420582; doi:10.1038/s41467-019-09236-4)
Supplement: Supplementary file 1 — Supplementary Information [file 41467_2019_9236_MOESM1_ESM.pdf]

Supplementary Information

Life habits and evolutionary biology of new two-winged long-proboscid scorpionflies from Mid-Cretaceous Myanmar amber

Lin et al.

Supplementary Note 1 │ Phylogeny and Systematics of Mecoptera and Establishment of Dualulidae as a New Family........ 2
Historical Context........ 2
Phylogenetic Procedures and Methods........ 3
Phylogenetic Results........ 3
Supplementary Figure 1│Comparisons of eight, historically different results of phylogenetic analyses showing relationships among major holometabolan clades........ 4
Supplementary Figure 2 │Illustrations of the paratype of *Dualula kachinensis* gen. et sp. nov. 5
Supplementary Figure 3 │ Details of the proboscis, calypter and genitalia for paratype *Dualula kachinensis* gen. et sp. nov. 6
Supplementary Figure 4 │ The paratype of *Dualula kachinensis* gen. et sp. nov. 7
Description of *Dualula kachinensis* Lin, Shih, Labandeira and Ren gen. et sp. nov........ 8
Supplementary Figure 5 │ Proboscis and genitalic details of paratype *Dualula kachinensis* gen. et sp. nov. 8
Evolution of Wing Venation in Mid-Mesozoic Scorpionflies........ 10
Supplementary Figure 6 │ Comparison of forewing venation in *Vitimopsyche, Lichnomesopsyche* and *Dualula*........ 11
Future Studies of Pseudopolycentropodidae, *Parapolycentropus* and Dualidae........ 13

Supplementary Note 2 │ Mouthparts of Long-Proboscid Mecoptera........ 14

Supplementary Note 3 │ *Dualula* Mouthpart Structure........ 17
Supplementary Figure 7 │ Proboscis details of *Pseudopolycentropus janeannae*........ 17
Supplementary Figure 8 │ Details of the proboscis base and other mouthpart elements of *Parapolycentropus* spp........ 18
Supplementary Figure 9 │ Proboscis of *Parapolycentropus paraburmiticus*........ 20

Supplementary Note 4 │ Feeding Processes and Food Sources of *Parapolycentropus* and Dualulidae........ 20
Associations with Gymnosperms........ 21
Supplementary Figure 10 │Measurements and distribution of *Cycadopites* sp. pollen grains adjacent to *Parapolycentropus paraburmiticus*........ 22
Possible Associations with Angiosperms........ 22
Supplementary Figure 11 │ *Tropidogyne* spp. and five angiosperm flower morphotypes with attached stamens from Myanmar amber........ 23

Supplementary Note 5 │ Hind-Wing Reduction in Mid-Mesozoic Insects, Pseudopolycentropodidae, *Parapolycentropus* and Dualulidae........ 24

Supplementary Note 6 │ Genitalia Structure in Mesopsychidae, Pseudopolycentropodidae, *Parapolycentropus* and Dualulidae........ 26
Supplementary Figure 12 │ Male genitalia of *Pseudopolycentropus janeannae*........ 26
Supplementary Figure 13 │ Male genitalia of *Lichnomesopsyche daohugouensis*........ 27
Supplementary Figure 14 │ Male genitalia of *Epicharmesopsyche pentavenulosa*........ 28

Supplementary Note 7 │ Reproductive Biology of *Parapolycentropus* and Dualulidae........ 29
Supplementary Figure 15 │ Three entombed swarms of *Parapolycentropus* spp........ 29
Supplementary Figure 16 │ Copulating *Parapolycentropus paraburmiticus*........ 30

Supplementary Table 1........ 33

Supplementary References........ 34

Supplementary Note 1 │ Phylogeny and Systematics of Mecoptera and Establishment of Dualulidae as a New Family

Historical Context

During the past half century, numerous phylogenetic analyses that have attempted to resolve the phylogenetic relationships among the major lineages within Holometabola[1–22]. Hennig, in his seminal work in 1969, presented a phylogeny of Holometabola that included Neuroptera and Mecopteroidea, the latter of which involved an investigation of the relationships among the mecopteroid groups of Mecoptera, Diptera and Siphonaptera, and their phylogenetic connection to Amphiesmenoptera (Trichoptera + Lepidoptera)[1]. At the time these analyses were based entirely on morphology; with character matrix construction and character sampling often incomplete, notably in Hennig's[1] and Boudreaux's[2] studies. Nevertheless, their efforts had substantive implications for later research. For example, Hennig placed Mecoptera and Diptera as a sister-group to Amphiesmenoptera, and Siphonaptera was phylogenetically close to Neuroptera and distant from Mecoptera[1] (Supplementary Fig. 1a). The study of Kristensen (1999)[3] partly supported the Hennig's earlier results[1]. Nevertheless, Boudreaux provided evidence for Siphonaptera as the sister-group to Diptera, and together with Mecoptera, formed a monophyletic group[2] (Supplementary Fig. 1b).

With the development of molecular phylogenetic approaches, molecular character matrices supplanted morphological data and became the mainstay for analyses of Holometabola, especially for those analyses that only contained extant groups. Later, phylogenetic analysis typically involved a total evidence approach that included morphological and molecular data, such as analyses by Beutel and colleagues[4], which provided a result similar to that of Boudreaux[2]. In two other analysis employing a total evidence approach, Diptera was proposed as allied with Amphiesmenoptera, and Mecoptera was a sister-taxon of Siphonaptera[5,6] (Supplementary Fig. 1g). These two analyses were based on 18S ribosomal RNA[5] and mitochondrial genome[6] data, respectively. By contrast, Chalwatzis and colleagues proposed that Diptera derived from within a clade consisting of Mecoptera, Siphonaptera and Neuropteroidea, and that Amphiesmenoptera was the basal member in these three groups[7] (Supplementary Fig. 1d). This phylogenetic topology[7] was at variance with views that are more recent.

Whiting was the first to attempt a phylogenetic analysis of extant Mecoptera with multiple gene data, Based on his results[8] (Supplementary Fig. 1f), Mecoptera is a paraphyletic group, with Boreidae the sister-group of Siphonaptera, and the position of Diptera lay outside of Mecoptera and Siphonaptera. As Whiting's data[8] included only extant groups, his phylogeny resulted in gaps that lacked fossil taxa. Similarly, the subsequent analysis by Song et al.[6], which used mitochondrial genome data for their phylogenetic analysis of Holometabola, confirmed that mitochondrial genome data was insufficient for deciphering deep relationships within Holometabola[6]. Currently, most results have (i) supported Mecoptera and Siphonaptera as forming a clade that has a close relationship to Diptera; (ii) determined that Amphiesmenoptera is a monophyletic group; and (iii) relegated Neuroptera as a significantly distant group from Mecoptera, Siphonaptera, Diptera, Trichoptera and Lepidoptera (Supplementary Fig. 1c). Of these studies, some contain only morphological matrices[9,10], others molecular matrices[11–16], and more recently total evidence[17–19]. However, the majority of these analyses has focused only on extant groups[20], and consequently has ignored the importance of fossil records. Such a focus has resulted in the lack of exploration in the relationship between extinct and extant groups. By integrating extinct and extant groups into a phylogenetic analysis[21], the paraphyly of Mecoptera is upheld, rendering Diptera and Siphonaptera as lineages within Mecoptera and the existence of a close relationship between Amphiesmenoptera and the most basal Mecoptera lineage, Kaltanidae[21] (Supplementary Fig. 1h).

The results of our research (Fig. 1; Supplementary Fig. 1i) indicate Amphiesmenoptera and Thaumatomeropidae are sister groups, and basal Diptera and Siphonate are close to the new clade

Dualulidae and *Parapolycentropus*. Based on our analysis, we conclude that the subgroups of Antliophora – Mecoptera, Diptera and Siphonaptera – are paraphyletic. This result generally is similar to the conclusion of Wheeler et al.[20] (Supplementary Fig. 1e), but differs from other studies, including Ren et al.[21], that result in several clades. Our new results are different in four ways. First, basal Diptera and Siphonaptera occur within Mecoptera, rather than the sister group to Mecoptera. Second, Meropeidae and Eomeropidae are more basal than other extant families, compared to the basalmost position of Nannochoristidae in the previous studies. Third, basal Diptera has a close relationship with Siphonaptera. Fourth, Amphiesmenoptera and Thaumatomeropidae are sister-groups.

Phylogenetic Procedures and Methods

We carried out a phylogenetic analysis to explore the taxonomic position of Dualulidae and to clarify the phylogenetic relationships between the two-winged mecopteran taxa and other scorpionflies, including the long-proboscid clade Aneuretopsychina. Because of an absence of well-preserved body features in most specimens of the extinct families, we were able to include in the analysis only some of the available body characters. The list of characters (Supplementary Data 1) included features of the mouthparts, head, thorax and legs (characters 0–13); features of the wings such as vein number, wing shape and venation (characters 14–46); and features of the abdomen and genitalia (characters 47–50). All 51 characters were used in the phylogenetic analysis. The character selection was partly attributable to the characters used in the phylogenetic analyses of Ren et al. (2009)[21] and Lin et al. (2016)[22]. Thirty-four genera or subfamilies comprising six extant families of Mecoptera, Siphonaptera, and seven genera of basal Diptera with complete or nearly complete preserved wings and bodies were selected as ingroups for the analyses (Fig. 1).

Type genera were used for most families of Mecoptera in the analyses. However, if the type genera for some families lacked a full or nearly full complement of features, such as an incomplete fore- or hind wing, we employed non-type genera that were more complete morphologically to represent the full or fullest character set achievable for the given taxon. For example, the family Aneuretopsychidae is best represented not by *Aneuretopsyche*, but rather *Jeholopsyche*, the only genus with well-preserved body and mouthpart features for this family. For basal Diptera, selection of genera originated from the tree of Blagoderov et al. (2007)[23]. The earliest fossil records of siphonate proboscides – *Permithone* as Permithonidae)[24–26] and Amphiesmenoptera[1,13,27,28] – were selected as outgroups. The selection of outgroups was based on the phylogenetic results of the Misof et al. (2014) study[13] and the analysis of Wiegmann et al. (2009)[14] that showed relationships among dipterans, siphonapterans, mecopterans and related taxa, including stem-groups. We chose the type genus *Permithone* of Permithonidae (Neuroptera) as the root of the tree, and a character-state matrix of 37 taxa and 51 morphological characters with two or more character states (Supplementary Data 2).

The character-state matrix was entered into WinClada (Version1.00.08)[29]. For a tree search we used a heuristic search method, with options set to hold 10,000 trees, 1000 replications, 100 starting tree replications, and a multiple TBR + TBR search strategy. All characters were treated as unordered and equally weighted. Missing characters were coded with a question mark and inapplicable characters with an em dash. Multi-state characters were denoted as a “0+1” mark. Parsimony analyses were performed by NONA (Version 2.0)[30] using an exhaustive search option and bootstrap support values from 1000 replications are presented as numbers under branch nodes.

Phylogenetic Results

All trees indicate that Mecoptera are a paraphyletic group, and the two most basal taxa are Thaumatomeropidae[22] consisting of *Thaumatomerope*[31,32] and Kaltanidae[33] represented by *Altajopanorpa*[33,34]. Thaumatomeropidae exhibits a close relationship to Amphiesmenoptera (Fig. 1a), but,

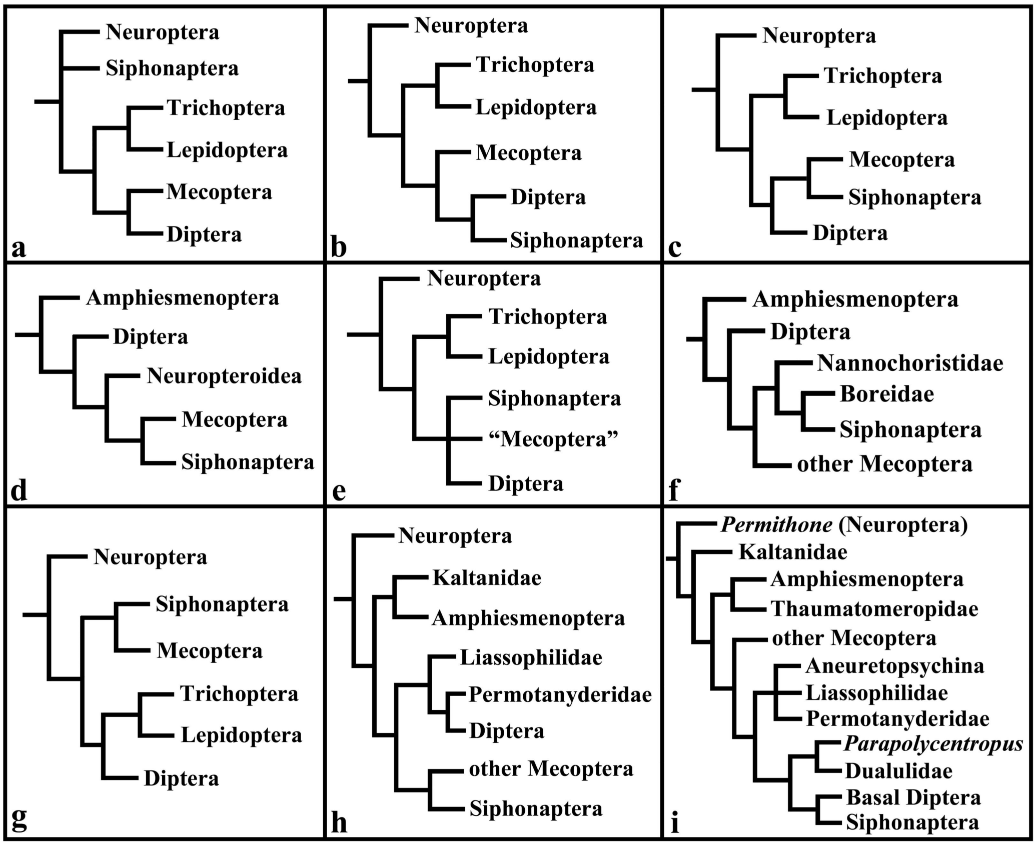

Supplementary Figure 1 │Comparisons of eight, historically different results of phylogenetic analyses showing relationships among major holometabolan clades. The included clades are Neuroptera, Amphiesmenoptera (Trichoptera, Lepidoptera), and Antliophora (Mecoptera, Diptera, Siphonaptera) and subgroups. (**a**), Phylogenetic tree abstracted from Hennig (1969)[1]. (**b**), Phylogenetic tree abstracted from Boudreaux (1979)[2]. (**c**), Phylogenetic tree abstracted from Misof et al. (2014)[13] and Wiegmann et al. (2009)[14]. (**d**), Phylogenetic tree abstracted from Chalwatzis et al. (1996)[7]. (**e**), Phylogenetic tree abstracted from Wheeler et al. (2001)[20]. (**f**), Phylogenetic tree abstracted from Whiting (2002)[8]. (**g**), Phylogenetic tree abstracted from Song et al. (2016)[6]. (**h**), Phylogenetic tree abstracted from Ren et al. (2009)[21]. (**i**), Phylogenetic tree abstracted from Fig. 1 of this study.

---

this link is established only on two homologous characters, defined by fewer crossveins (Character 41:1) and lack of pterostigma in the forewings (Character 43:1). A revision of these taxa would be in order with use of additional, better-preserved fossil material than at present. These two nominal mecopteran groups, with Amphiesmenoptera, serve as the sister group of all other Mecoptera, though the basal position of Thaumatomeropidae and Kaltanidae is not secure. Meropeidae[35] consists of *Burmomerope*[36] and Eomeropidae, with *Eomerope* as the basalmost group of extant families[36,37]. Eomeropidae shares a sister-group relationship to other Mecoptera, basal Diptera, and Siphonaptera; however, this clade is supported by only one synapomorphic character, defined as leg pubescence forming regular, at least local encirclement features (Character 12:1). Nevertheless, the oldest fossil records of these two families are from the late Middle to early Late Triassic of Kyrgyzstan[32,38], a relatively late date when compared to the Late Permian for the oldest Aneuretopsychina[11,39,40]. This discrepancy in age may be attributable to the rarity of adequate fossil preservation or absence of discovery.

Two features render Belmontiidae, consisting of *Belmontia*[41] and its sister group, the balance of the clade, as forming a single lineage. One of these features is the unambiguous character of the presence of less than four anterior branches of the Sc vein in the forewing (Character 21:1); the other is the number of vein branches of the MP vein, a homologous character (Character 30:1). From this

relationship, the Permochoristinae[42] – equivalent to Permochoristidae[42,43] – was present during the late Permian, together with Sibiriothaumatidae that consisted of *Sibiriothauma*[44]. Previously, the earliest fossil record for *Sibiriothauma* was from the Late Jurassic of Russia[44]. *Sibiriothauma* forms a distinct clade with our data, confirming the homologous nature of the number of Rs branches and the relative level along the wing of the A2 vein ending versus the origination of the MP vein from the CuA vein in the forewings (Characters 26:1 and 39:1). This clade comprises the remainder of mecopteran taxa, seven basal dipteran lineages and Siphonaptera that are validated by three synapomorphic characters (Characters 22:1, 23:1 and 49:2) and one homologous character (Character 25:0). Characters 23 and 49 have parallelisms and reversals in the remaining branches. Parachoristidae[45] and the other four families of Panorpoidea display paraphyly. The other groups of this clade are monophyletic, supported by two synapomorphic features, a relatively short Sc vein in the forewings (Character 20:1) and hind wings (Character 44:1). There also are four homologous characters (Characters 19:1, 29:0, 41:1 and 49:1).

Curiously, six families of modern Mecoptera lack close relationships and occur with extinct families represented in the middle region of the trees (Fig. 1). These extant lineages evidently originated during the mid Mesozoic. Nannochoristidae[42] currently is recognized as the basalmost extant lineage[8], and has a sister-group relationship with extant Bittacidae[35] and extinct Robinjohniidae[46]. This relationship is supported by one synapomorphic character, the long sub-basal fusion of the MP and CuA veins in the forewings (Character 33:1). Two homologous characters (Characters 32:1 and 47:1) occur as well.

Toward the terminus of the tree, Aneuretopsychina are established as a paraphyletic group that includes Liassophilidae and Permotanyderidae. Previously, Aneuretopsychina was considered a single,

---

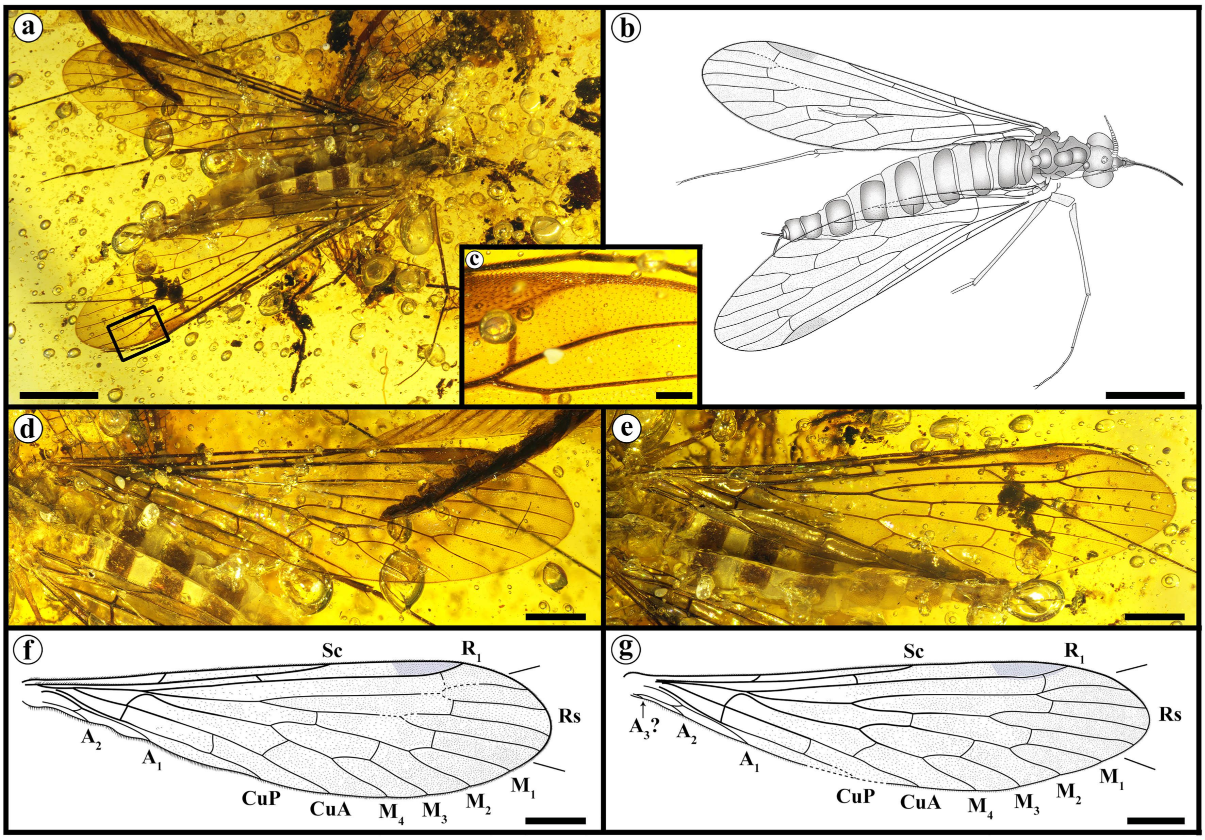

Supplementary Figure 2 │ Illustrations of the paratype of *Dualula kachinensis* gen. et sp. nov. This specimen is CNU-MEC-MA-2017016, a female. (**a**), Paratype in dorsal view. (**b**), Overlay drawing of the paratype in dorsal view. (**c**), Details of marginal setae and membrane of the right forewing. (**d**), Left forewing. (**e**), Right forewing. (**f**), Line drawing of left forewing. (**g**), Line drawing of right forewing. Scale bars represent 2 mm in (a) and (b), 1 mm in (d)–(g), and 0.2 mm in (c).

independent clade[21], buttressed by two explicit characters, siphonate mouthparts (Character 4:1), and maxillary palpi that were short and adpressed to the base of the proboscis or alternatively lost as a multi-article structure (Character 5:1). However, the absence of complete body features of known Liassophilidae[47] and Permotanyderidae[26], especially involving presence or absence of a proboscis and other mouthpart elements, is an issue. The presence of a proboscis in these two lineages may expand the monophyly of the three genera currently constituting Pseudopolycentropodidae[48,49] (Supplementary Table 1), presently established only by three homologous features (Characters 12:2, 25:0 and 46:0). These three features yield the same result for the other three families of Aneuretopsychina (Characters 15:0, 16:0 and 32:1) consisting of Dualulidae, *Parapolycentropus*[50], and Siphonaptera.

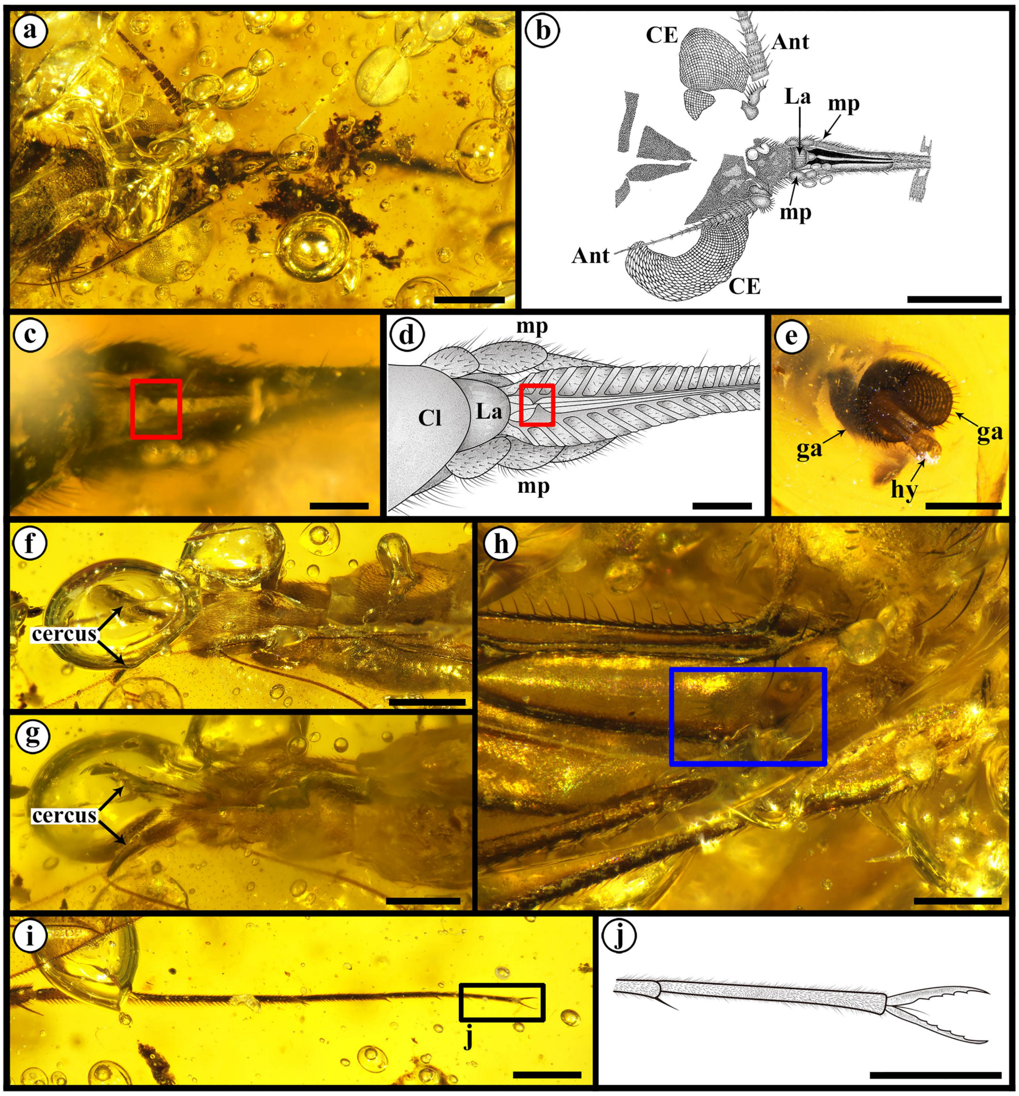

Supplementary Figure 3 │ Details of the proboscis, calypter and genitalia for paratype *Dualula kachinensis* gen. et sp. nov. This specimen is CNU-MEC-MA-2017016, a female. (**a**), Head and proboscis in dorsal view. (**b**), Combined camera lucida and overlay drawing of the head and mouthparts; the ovoidal and circular structures are bubbles that occlude structural details. (**c**), Proboscis base. (**d**), Line drawing of proboscis base. (**e**), Proboscis cross-section – revealing outer galeal food tube and inner hypopharyngeal salivary duct – that is uniquely exposed at the amber surface. (**f**), Female genitalia in dorsal view. (**g**), Female genitalia in ventral view. (**h**), Right hind wing (calypter) in dorsal view. (**i**), Left hind leg in dorsal view. (**j**), Line drawing of telotarsus and claws of left hind leg. Red rectangles in (c) and (d) indicate the distinctive valve regulating the salivarium chamber at the hypopharyngeal base. Scale bars represent 0.5 mm in (a), (b), (f), (g) and (i); 0.1 mm in (c)–(e); and 0.2 mm in (h) and (j).

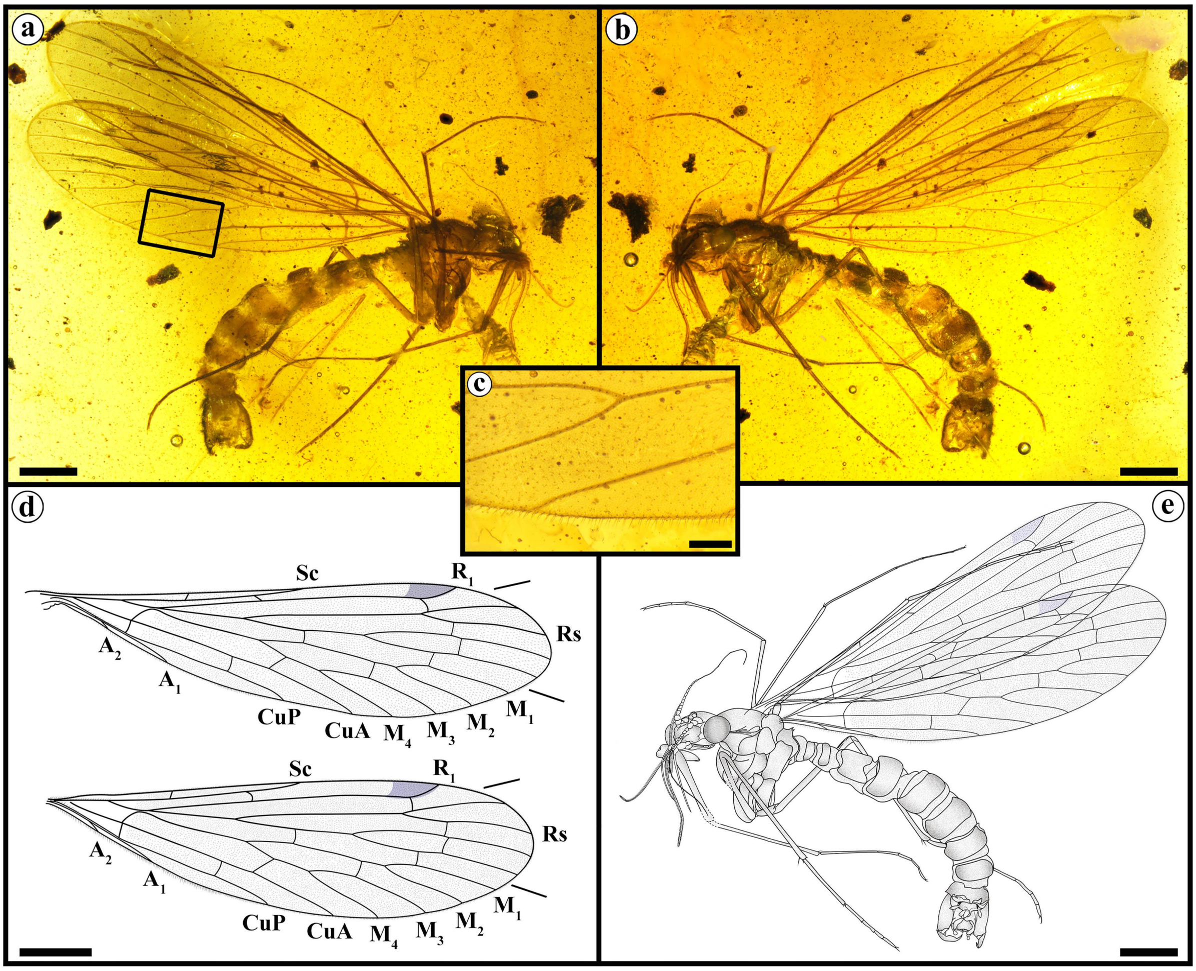

Supplementary Figure 4 │ The paratype of *Dualula kachinensis* gen. et sp. nov. This specimen is CNU-MEC-MA-2017017, a male. (**a**), Paratype in right lateral view. (**b**), Paratype in left lateral view. (**c**), Details of setae on the left forewing membrane margin. (**d**), Line drawing of forewings; above is the right and below is the left forewing. (**e**), Overlay drawing of (b). Scale bars: 1 mm in (a), (b), (d) and (e); 0.2 mm in (c).

Seven basal dipteran taxa constitute a clade that is supported by five synapomorphic characters. The first character is that only the mesothorax is structurally robust or alternatively none of the three thoracic segments is structurally robust (Character 8:1). Second, only one pair of wings is present or alternatively both pairs are lost (Character 14:1).Third, heteronomous hind wings are reduced in size and modified into halteres or haltere-like structures (Character 15:2). Fourth, thickened setae are present along the margin of the forewing (Character 18:1). Fifth, the hind wings are vestigial (Character 45:1). Additionally, two homologous features (Characters 11:1 and 49:0) are present. Three definite features support the clade of basal Diptera and Siphonaptera. They are i), absence of a membranous area between the mesopleura and metapleura (Character 10:1); ii), partial axillary sclerites occurring at the base of the forewings (Character 17:1); and iii), hind wings are modified into halteres (Character 45:2). By contrast, seven genera of basal dipterans and Siphonaptera are not adequately separated and display extensive paraphyly. Due to the wingless condition of Siphonaptera, most characters used in our analysis are not applicable. Therefore, the position of Siphonaptera cannot be well- established. The resulting tree 24 (Fig. 1b) essentially is the same as the strict consensus tree (Fig. 1a), with two obvious differences. The first difference is that Parachoristidae[45] and the four families of Panorpoidea – Dinopanorpidae[51], Orthophlebiidae[35], Panorpidae[52] and Panorpodidae[48] – show collapse of three nodes resulting in these families becoming paraphyletic in the consensus tree (Fig. 1a). Second, there are no

synapomorphic characters distinguishing the basal dipterans and Siphonaptera, resulting in extensive paraphyly in the consensus tree. In addition, only one synapomorphic character (Character 10:1) supports this clade.

Description of *Dualula kachinensis* Lin, Shih, Labandeira and Ren, gen. et sp. nov.

This description is based mostly on the holotype specimen, but also is partly based on two paratype specimens that especially involve details of the head, proboscis and genitalia. (See Figs. 1–3 and Supplementary Figs. 2–5).

Head and mouthparts. Head triangular in dorsal view; mouthparts prognathous. Three ocelli present, and several long and robust setae on the vertex (Fig. 1i; Supplementary Fig. 5a,b). Distinctive carinae between clypeus and antennae. Antenna filiform, with many articles; scape tubular and large; pedicel slightly funnel-shaped; flagellum with 20 flagellomeres, the basal 10 flagellomeres much thicker and shorter than the remaining 10, among them flagellomeres 1–6 larger than flagellomeres 7–10; flagellomeres 11–20 long and thin, almost long rectangular; and last two flagellomeres much shorter than flagellomeres 11–18. Compound eyes large, oval and widely separated. Proboscis long and narrow; (Fig. 1a,b,d,i; Supplementary Fig. 5a,b); male shorter than female (Supplementary Fig. 2a,b); lacking apical pseudolabellae, covered with dense setae or smaller microtrichia and displaying encircling annuli or bands. Labrum relatively small, triangular and apex slightly round. Proboscis divided into three elements:

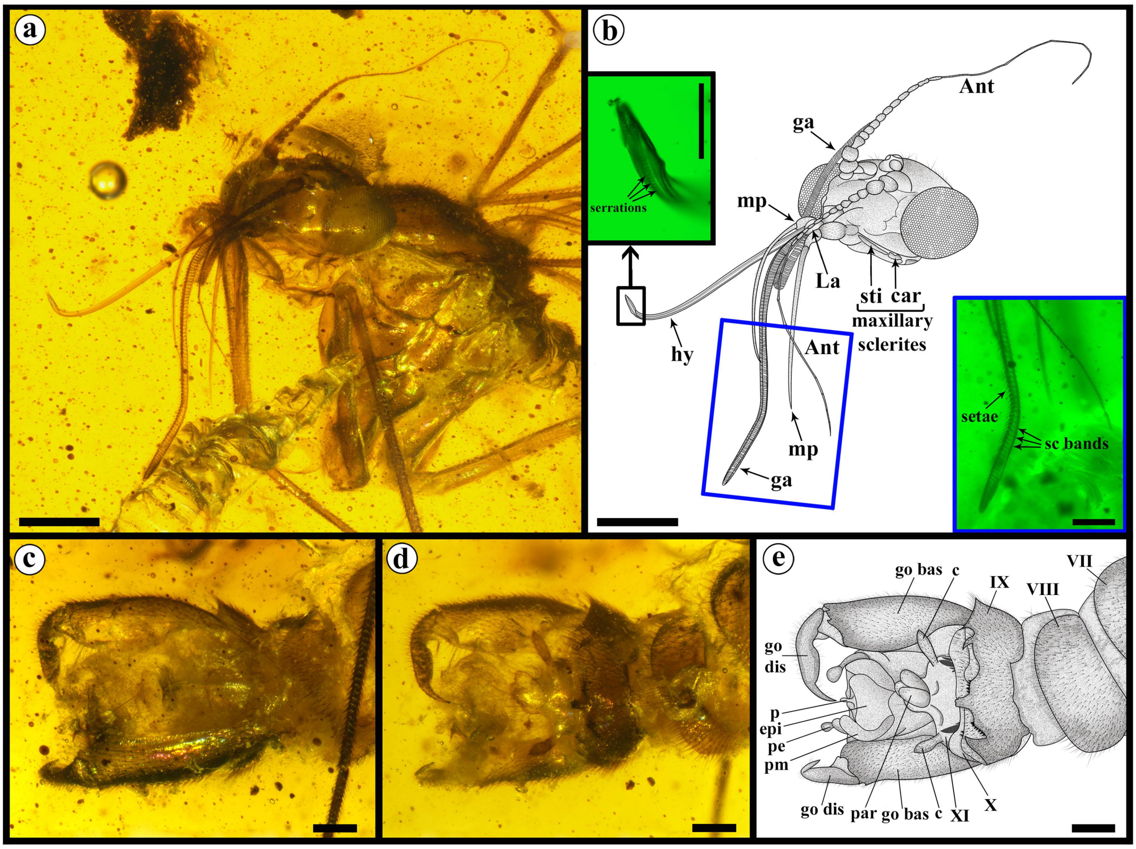

Supplementary Figure 5 │ Proboscis and genitalic details of paratype *Dualula kachinensis* gen. et sp. nov. This specimen is CNU-MEC-MA-2017017, a male. (**a**), Head and proboscis in left lateral view. (**b**), Line drawing of head and proboscis in (a), and enlargement of hypopharynx tip (upper left) and galeae (lower right) under green epifluorescence. (**c**), Male genitalia in ventral view. (**d**), Male genitalia in dorsal view. (**e**), Line drawing of male genitalia in (d). Abbreviations: **car**, cardo; **ga**, galea; **hy**, hypopharynx; **mp**, maxillary palp; **sti**, stipes; **c**, cercus; **epi**, epithallus; **go bas**, gonocoxa basistylus; **go dis**, gonocoxa dististylus; **p**, penis; **par**, paraprocts; **pe**, penunci; **pm**, paramere; and **tVI**–**tIX**, sixth to ninth terga. Scale bars represent 0.5 mm in (a) and (b); 0.1 mm in hypopharynx tip of (b), 0.2 mm in galeae tip of (b); and 0.2 mm in (c)–(e).

a hypopharynx and a pair of encompassing galeae. Maxillary palp originating from palpifer of stipes connected to cardo; these maxillary sclerites located on either side of central long, labial sclerite, the mentum. Maxillary palp with three articles, the second article widest and terminal article much longer and narrower than other two, especially extended in male with length ca. two-thirds of galea and hypopharynx (Supplementary Fig. 5a,b). Each galea a half tube in lateral section (Supplementary Fig. 3e), with sclerotized and setose ring bands on outer surface, but inner-surface smooth and lacking setae; galeae sutured to each other, forming a food channel that encompasses the hypopharyngeal salivary duct. Salivary duct deployed as a narrow tube emerging from a valved salivarium chamber (hypopharyngeal pump) under labrum (Supplementary Fig. 3a–d), positioned rectilinearly from labrum to proboscis tip (Fig. 1g–i; Supplementary Figs. 3c–e; 5a,b); many serrations present on ventral aspect of salivary duct (Fig. 1g,h; Supplementary Figs. 3e, 5b). Galea more flexible than hypopharynx; both elements not bound to each other (Fig. 1g; Supplementary Fig. 5b).

Thorax and Legs. Pronotum and metanotum small and similar to each other; mesonotum relatively large (Fig. 2a,b). Scutum and scutellum distinct on mesonotum and metanotum. Legs slender and entirely covered with pubescence (Supplementary Fig. 3i,j) but not arranged into annulus. Femora elongate; tibiae long and slender, with at least one apical spur. Tarsi of five segments; the first segment longest and nearly the same length as rest of segments, last two segments shortest and same in size and shape; pretarsus with two claws and one long bristle between them, the inner side of each claw bears four sharp tooth-like structures, all similar in size and shape (Supplementary Fig. 3i,j).

Wings. Left forewing slender, right forewing very similar to left forewing (Fig. 1a–e; Supplementary Figs. 2, 4). Membrane delicate and tegula small (Fig. 2c–f). Sc relatively short; R_1 single and straight at the base, proximal to Rs_{1+2} bifurcation, slightly curved near the margin; one sc-r_1 crossvein present, at the same level of or distal to Rs bifurcation. Pterostigma well preserved. Both Rs and M with four branches; Rs_{1+2} bifurcation distal to Rs_{3+4} bifurcation; one crossvein between R_1 and Rs_1, Rs_{1+2} and Rs_{3+4} respectively, and one rs_3-rs_4 crossvein present in male (Supplementary Fig. 4d); r_1-rs_1 slightly distal to Rs_{1+2} bifurcation, rs_2-rs_3 distal to rs_4-m_1; R from Sc proximal to M from CuA. M with four long branches, M_{1+2} forking distal to M_{3+4} bifurcation and much distal to Rs_{3+4} bifurcation, but proximal to Rs_{1+2}; one crossvein between M_{1+2} and M_3, very distal to M_{3+4}; M originating from CuA proximal to Rs from R_1; CuA, CuP. A_1 and A_2 single; A_1 relatively long and slightly distal to Sc_1, A_2 short; two crossveins between CuA and CuP, and one between A_1 and A_2. One calypter at the base of each forewing, with some thick, stiff setae along the margin of the apex (Fig. 2c–f; Supplementary Fig. 3h). Hind wing reduced to a minute, tubular-shaped lobe, with vestiges of some wing venation; relatively smooth, the length nearly the half of forewing calypter; also included an anterior lobe above the hind wing, and likely comprised of a tegula (Fig. 2c–e).

Abdomen and genitalia. In the female, abdomen elongate and tapering apically, with 11 segments (Supplementary Fig. 2a,b). T1 partly fused with metathorax, segments 8 and 9 much smaller than segments 2–7. The last two segments (10 and 11) very small and closely combined with each other. Tergites large and typical of scorpionflies, but sternites unknown due to preservation in both holotype and paratypes. Cercus originating from the eleventh segment and with two obvious articles, the first article slightly longer and thicker than second one, all articles covered with short and thick setae (Fig. 1f; Supplementary Fig. 3f,g).

In the male, abdomen much more slender than female, with nine visible segments. T1 small and fused somewhat with metathorax; the first and second segments much smaller than others. T9 enlarged, partly covering segments 10 and 11. Tergites wide, but sternites reduced and not visible because of obscuring light color (Supplementary Fig. 4a,b,e). Male genitalia well preserved; tergites 10 and 11 relatively small; a pair of short cerci and an anal orifice originating from segment 11 (Supplementary Fig.

5d,e); paraprocts almost entirely covering the anal orifice. The ninth sternite (S9) enlarged and occupying nearly entire venter of gonosomite; clasper (or gonocoxa) large and robust, originating from sternite 9 and divided into two distinct segments – basistylus and dististylus. Basistylus immovable and combined with S9; dististylus hook-shaped and somewhat expanded in its middle section but tapering, length about two thirds of basistylus. Aedeagus consists of three parts, epiphallus, penunci and paramere, and penis; epiphallus infundibulate and almost covers the penis; parameres slender and rodlike, but penunci much smaller and tubular (Supplementary Fig. 5d,e). Several long and thick setae or bristles cover the ninth tergum tIX, paraprocts, cercus and clasper (Supplementary Fig. 5c–e).

Evolution of Wing Venation in Mid-Mesozoic Scorpionflies

In Neuroptera, three functionally two-winged species were described from the Cretaceous, potentially providing additional insight into the evolutionary process of wing reduction. A mantispid species, *Mantispidiptera enigmatica* (Mantispidae), from Upper Cretaceous (Turonian) amber of New Jersey[53], displays a two-winged condition with highly reduced hind wings. Similarly, the two-winged lacewing, *Dipteromantispa brevisubcosta* (Dipteromantispidae), was described from the Lower Cretaceous (latest Barremian–earliest Aptian) Yixian Formation of Liaoning, in Northeastern China[54]. This specimen displayed two normal forewings while hind wings were modified into small halteres. Lastly, the species *Pedanoptera arachnophila* (Mesochrysopidae) was described from Myanmar amber[55]. This species also exhibited highly reduced hind wings, but unlike the other taxa, it retained a few discernable longitudinal veins[55]. Mid-Mesozoic hind-wing reduction also independently appeared in several lineages of Mecoptera. Such hind-wing specialization is not an accidental phenomenon, but rather represents a cascade of evolutionary developmental processes that was repeated in several, major insect lineages such as Neuroptera and Mecoptera.

The wing venation of the Dualulidae is similar to that of Mesopsychidae, particularly *Vitimopsyche*[56] and *Lichnomesopsyche*[57]. Although a distinct system of vein nomenclature historically has been used to describe mesopsychid venation, the six principal venational similarities of the Dualulidae and Mesopsychidae are the following. First, the Sc vein of most species has only one anterior branch, excluding *Vitimopsyche torta*[56]. Second, the Rs and MA veins have two branches, and the MP vein has four branches. Third, the CuA and CuP single, unbranched veins are associated with the two anal veins. Fourth, the stem of the MP vein distinctly curves at the base of the wing and forms a near right angle, a condition occurring in *Dualula* and most species of *Vitimopsyche*. Fifth, the origin of the MP vein from the CuA vein is at the same level lengthwise in the wing as the first cua-cup crossvein, seen in *Dualula* and *Vitimopsyche*. Sixth, the origin of the MP vein from the CuA vein is almost at the same level as the origin of the Rs+MA vein from the R_1 vein, a feature found in *Dualula* and *Lichnomesopsyche*.

By contrast, the Dualulidae differs from *Vitimopsyche* and *Lichnomesopsy*che in the following six ways. First, the anal field of the wing is distinctly narrow in Dualulidae, compared to a much broader field found in Mesopsychidae. Second, the Sc is relatively short, and much closer to the MP_{1+2} bifurcation in both families than in Dualulidae. Third, the posterior margin of the wing at the CuP vein apex lacks an embayment in Dualulidae and in two species of *Vitimopsyche* (Supplementary Fig. 6a,g). Fourth, the Rs bifurcation is considerably distal to the MA vein and the stem of MA and MA_1 does not form an S-shaped vein. Fifth, the MP_{1+2} and MP_{3+4} bifurcations are considerably more distal to the MP vein bifurcation; and the MP_{1+2} much more distal to the MP_{3+4}. Sixth, the 1A and 2A are relatively short and contact the wing margin nearly at the same level as the separation of the Rs+MA from the R_1 vein (Supplementary Fig. 6.)

The wing and body size of Dualulidae are substantially smaller than most species of Mesopsychidae. We examined three specimens of Dualulidae, two females and one male, and 16 specimens of Mesopsychidae, five female, three male and eight unassigned to sex. The Dualulidae forewing length is 9.4 mm for females and 7.1 mm for males; similarly, their body length is 8.26 mm for females and 7.62

mm for males. Nevertheless, forewing and body length in Mesopsychidae for well-preserved specimens suggest that the size of most Mesopsychidae was small to medium for a mid-Mesozoic insect. For Mesopsychidae, the length of the right forewing ranges from 5.73 mm to 29.02 mm, with an average of 21.35 mm (data includes specimens with one preserved forewing). Except for most species of Permian *Permopsyche*[58] and some species of *Mesopsyche*[41,58,78], the forewing length of all Mesopsychidae species is greater than 21 mm and is 2.23–2.96 times as long as Dualulidae. Moreover, the body size of *Mesopsychidae* is approximately 23.15 mm, 2.46–3.26 times as long as Dualulidae. However, the ratio of forewing length to body length (excluding proboscis and antennae) are minimally different between Mesopsychidae and Dualulidae, ranging from 0.96 to 1.48 and 0.93 to 1.14 respectively.

These data indicate that whereas wing and body size differed considerably between Mesopsychidae and Dualulidae, body shape, or aspect ratios, remained relatively stable. (Supplementary Data 4 and 5.) *Dualula* is very similar to *Parapolycentropus* except for six significant morphological differences. First, unlike *Parapolycentropus*, tergum VIII of males is present in *Dualula*, but is fused with tergum IX or lost in

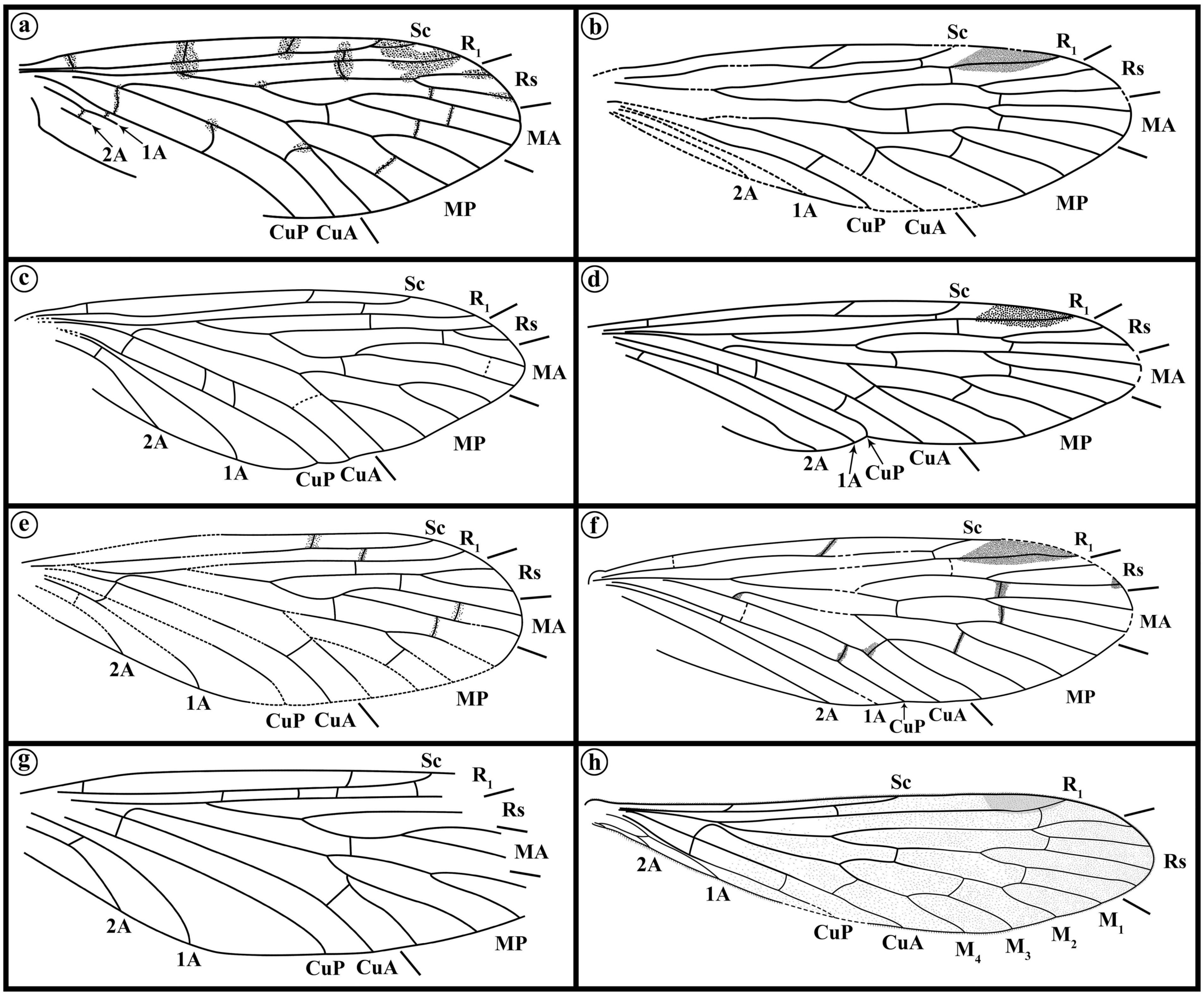

Supplementary Figure 6 │ Comparison of forewing venation in *Vitimopsyche*[56], *Lichnomesopsyche*[57] and *Dualula*. (**a**), Line drawing of *Vitimopsyche torta*[56]. (**b**), Line drawing of right forewing of *Lichnomesopsyche prochorista*[22]. (**c**), Line drawing of *Vitimopsyche kozlovi*[57]. (**d**), Line drawing of *Lichnomesopsyche gloriae*[57]. (**e**), Line drawing of *Vitimopsyche pristina*[22]. (**f**), Left forewing line drawing of *Lichnomesopsyche daohugouensis*[22] (CNU-MEC-MA-2015011P/C). (**g**), Line drawing of *Vitimopsyche pectinella*[59]. (**h**), Right forewing line drawing of *Dualula kachinensis* gen. et sp. nov. (Paratype, CNU-MEC-MA-2017016, a female). Except (h), all others are redrawings based on the published line drawings. Drawings are not to scale.

Parapolycentropus. Second, the microstructure of the male genitalia is different in several respects. The gonostylus of *Dualula* is poorly developed, but upturned in *Parapolycentropus*. The gonocoxa dististylus is inflated in its central part in *Dualula*, in contrast to the tapering condition in *Parapolycentropus*. The gonocoxa basistylus in *Dualula* is olive shaped and smooth, in contrast to a V-shaped structure housing a spine in *Parapolycentropus*. Although the shape of tergum IX is distinct in these two groups, the distal tergal edge consists of three or four obvious, cusp-shaped protuberances in *Dualula*, but is composed of a row of denticles in *Parapolycentropus*. In *Dualula* the penis and subsidiary structures are present, while conspicuously absent or invisible in *Parapolycentropus*. Third, the antenna of *Dualula* consists of 20 flagellomeres, of which flagellomeres 1–7 are nearly trapezoidal in shape, whereas in *Parapolycentropus*, only 16 flagellomeres are present and the basal five flagellomeres appear tapered. Fourth, there are several proboscis-related differences between these two taxa. The proboscis is considerably more narrow and slender in *Dualula* than most *Parapolycentropus*, which have a comparatively more robust proboscis. The ratio of length to the width of the proboscis is 32.3 in female and 16.54 in male *Dualula*, in contrast to an average of 14.03 in *Parapolycentropus* regardless of gender. Moreover, the third segment of the maxillary palp is not obviously different in length between male and female in *Parapolycentropus*. However, it is considerably extended in *Dualula* males and less so among *Dualula* females. Notably, a distinctive hypopharyngeal valve occurs in *Dualula*, a condition evidently missing in *Parapolycentropus*. Fifth, in *Dualula* each leg bears two tarsal claws terminally and a thick and long seta in the middle; in distinction, the tarsal claws of *Parapolycentropus* are highly variable, wherein even one individual can express differences in claw form across its prothoracic, mesothoracic and metathoracic legs. In *Dualula*, the tibiae of the metathoracic legs lack sexually dimorphic, robust setae; in *Parapolycentropus*, there is a row of nine thick and sharp setae on the tibiae. Sixth, there are several important differences in forewing venation between these two taxa. In *Dualula*, the Sc vein is about two times as long as it is in *Parapolycentropus*; in similar fashion, in *Dualula* the Sc vein displays one, short anterior branch in contrast to the absence of branching in *Parapolycentropus*. *Dualula* lacks a crossvein between the R_1 and C veins, and in *Parapolycentropus,* it is replaced by a sc–r1 crossvein. In *Dualula*, an R vein ends at the C vein in a position considerably proximal to the Rs_{1+2}; by contrast, in *Parapolycentropus* this vein ends at the same level or is only in a slightly proximal position. The M vein in *Dualula* has four, long branches from an unmerged vein stem, whereas in *Parapolycentropus* the stem of the M vein has a curved course and forms an almost right angle. *Dualula* lacks a dc cell, which is present in *Parapolycentropus*. The anal field is relatively broad in *Dualula*, with at least two (or three) branches of the anal vein, in contrast to a narrow anal field in *Parapolycentropus* and only one or two branches of the anal vein. The CuP and anal veins are longer in *Dualula* than in *Parapolycentropus*. In *Dualula*, the CuP vein occurs at the same level as the bifurcation of the M; by contrast, in *Parapolycentropus* the CuP vein is present at a position much more distal to the M vein bifurcation. As well, the wing shape varies between the two genera. The ratio of wing length to width is 3.83 in *Dualula* and about 3.0 in *Parapolycentropus*. While there are basic differences between *Dualula* and *Parapolycentropus*, other commonalities in characters and life habits may warrant future placement of *Parapolycentropus* in the Dualulidae or at least as a sister-group to this family.

Many of these morphological differences in wing characters are under the control of homeotic (*Hox*) genes. It long has been known that such genes determine the fate of the fly's second and third thoracic segments, and these genes are particularly involved in wing development[60,61]. The Hox gene *Ultrabithorax* (*Ubx*), is segment specific, avoids the forewing as a target, and expresses wing disc cells of the third segment of the larva, from which the hind wing normally develops. When *Ubx* is expressed at elevated levels in the third thoracic segment throughout larval development, it causes haltere formation in the adult[62]. In this ensuing cascade, the *apterous* gene also targets the *serrate* gene, which provides a ligand attachment to the receptor protein Notch. Notch produces the signaling molecule Wingless[63,64] that in the haltere activates only the anterior compartment, essentially deleting the posterior

compartment, resulting in a much abbreviated, elongate hind wing missing its posterior half[65]. Just in front of the anterior-posterior boundary, now essentially forming the posterior boundary of the hind wing, the production of another signaling molecule, Decapentaplegic (Dpp), activates a growth factor TGFß in conjunction with the presence of a second signaling factor, Hedgehog[66,67]. A target activated by the Hedgehog signal is *spalt*, a gene that typically is repressed by the *Ubx* protein, which with Dpp regulates haltere size[68]. Although the hind wings are very similar in *Parapolycentropus* and *Dualula*, *Ubx* and homologous Hox genes control expression of many of the above-mentioned morphological features.

Future Studies of Pseudopolycentropodidae, *Parapolycentropus* and Dualulidae

Previously, *Parapolycentropus* was a genus along with the other three genera that constituted Pseudopolycentropodidae (Figs. 5,6; Supplementary Figs. 8,9). In the resulting trees (Fig. 1) *Parapolycentropus*, however, is differentiated from these three genera of Pseudopolycentropodidae by body and wing structural characters examined in the phylogenetic analysis. Because of this new placement, it is suggested that additional studies of the systematics of core Pseudopolycentropodidae, *Parapolycentropus* and *Dualula* be made, with the goal of revising the classification of *Parapolycentropus*. It appears that three potential results for placement of *Parapolycentropus* are possible. First, *Parapolycentropus* is closely related to *Dualula* and it may be included as the second genus within the Dualulidae. Secondly, *Parapolycentropus* may belong to a new lineage, perhaps with a sister-group relationship to Dualulidae, where currently it is placed in the tree. Third, and more remotely, it may be reintegrated with core Pseudopolycentropodidae, perhaps as a sister group to the *Pseudopolycentropodes* + (*Pseudopolycentropus* + *Sinopolycentropus*) clade[35,49,77]. Several features contrast *Parapolycentropus* to other Mecoptera taxa, the most significant are wing features, particularly severe hind-wing reduction and features of forewing venation and shape[50] (Figs. 5; 6a,b; 7a,b; Supplementary Figs. 9a,b; 15). *Parapolycentropus* also shows considerable similarities to *Dualula*, particularly in mouthparts (cibarial and salivary pumps) and hind wing reduction (Figs. 1a,b,d,e; 2; 3; 6a,b; 7; Supplementary Figs. 2–5; 8; 9).

The phylogenetic analysis also revealed a close relationship between (*Parapolycentropus* + Dualulidae) + (Siphonaptera + basal Diptera) with Aneuretopsychina *sensu lato* in the next subjacent node. The Aneuretopsychina clade consists of Pseudopolycentropodidae + (Liassophilidae + {Permotanyderidae + [Aneuretopsychidae + ǁMesopsychidae + Nedubroviidaeǁ]}), the major lineages of which have highly variable times of origin. Of the four demonstrable long-proboscid clades, Mesopsychidae has a Late Permian time of origin in Australia[26] and a slightly younger occurrence from the Permian–Triassic boundary interval of Russia[58,78]. Nedubroviidae has a latest Permian time of origin[79] and an Early Triassic occurrence[40,79]. Pseudopolycentropodidae has an early Middle Triassic occurrence from France[76]; and earliest Aneuretopsychidae is from the Late Jurassic of Kazakhstan[85]. For the two possible long-proboscid clades, earliest Permotanyderidae occurs in the Late Permian of Australia[26]; and Liassophilidae has an earliest occurrence during the Early to Middle Triassic of France[81,124,125]. Although this disparity in the times of origin is attributable to a poor fossil record of terrestrial deposits, it provides evidence that Aneuretopsychina extends deep to the late Permian (Lopingian), had a first phase of early lineage diversification during the Early to Middle Triassic, and a subsequent phase of later lineage diversification in the Middle Jurassic to Early Cretaceous. Based on the trees in Figure 4, the Diptera would have had an origin during the Late Permian, although the earliest occurrence of a well-documented basal dipteran in Figure 1 is *Gallia*, assigned to Rhagionidae, of Early–Middle Triassic age[81,83].

Supplementary Note 2 │ Mouthparts of Long-Proboscid Mecoptera

Lineages within the traditional Aneuretopsychina – the assemblage or possibly clade that includes Mesopsychidae, Aneuretopsychidae, Pseudopolycentropodidae, Nedubroviidae and initially *Parapolycentropus* and the new family, Dualulidae, described in this report – are united by long-proboscid mouthparts[21]. These mouthparts display significant, important differences in structural details among these lineages. In Mesopsychidae, the proboscis is long, and consists of a closed, tubular siphon with an external surface randomly covered with thick setae[21], but lack the series of encompassing, sclerotized, annular rings reported here in Dualulidae. In addition to the absence of sclerotized bands, the mesopsychid proboscis lacks external ornamentation such as transverse ridges that define the Aneuretopsychidae[21]. Pseudopolycentropodidae by contrast contains diminutive sclerotized bands with microtrichia, which often give the appearance a smooth, featureless surface in compression fossils[49]. Ovoidal labial pads, the pseudolabellae, occur prominently at each side of the proboscis terminus in Mesopsychidae, are structurally quite different in Aneuretopsychidae, and absent entirely in Pseudopolycentropodidae[21,49,50] and Dualulidae. Individual mouthpart elements of the Mesopsychidae proboscis of one specimen were observed as separated into two, perhaps three, elongate structures that probably represent tongue-and-groove interlocking features[21] that keep the galeal walls of the proboscis intact and tubular. By contrast, the most distinguishing proboscis characteristic of Aneuretopsychidae is the distinctive, transverse, annular ridges and dense setae or microtrichia arranged perpendicularly along these ridges on the external proboscis surface[21,39,83]. Aneuretopsychid pseudolabellae consist of a distinctive, large, U-shaped pseudolabellum that probably increases the contact area with the substrate, allowing for more conducive absorption of surface fluids. Other elements of the mouthpart complexes of Aneuretopsychina are poorly known. However, the mesopsychid maxillary palp has three robust articles, apparently is positioned abutting along the labrum, and has a third segment curved in shape and slightly shorter than other two. Aneuretopsychidae evidently houses a cibarial pump below the clypeus[21,83] that provides for the flow of incoming food along the proboscis.

The directional deployment of the Aneuretopsychina proboscis is important for understanding the life habits of these lineages. For Aneuretopsychidae the proboscis was deployed in a backwardly directed, opisthognathous position. For Pseudopolycentropodidae and Dualulidae, the proboscis was directed in a forwardly jutting prognathous position, as in Mesopsychidae[21,49,50]. These two proboscis orientations are similar to mouthpart placement in sternorrhynchan Hemiptera and Lepidoptera, and contrast with the mostly hypognathous position of mouthparts in extant Mecoptera[21,84]. For Nedubroviidae, because of poor preservation of the proboscis, the most recognizable feature is the base of the proboscis, which includes a triangular and enlarged labrum, possibly partly fused with the clypeus that supports an extended rostral base and a robust, prognathous proboscis[79]. These proboscis orientations indicate that their proboscides were used for different purposes and can be contrasted to other, contemporaneous, long-proboscid lineages. Various lineages of Aneuretopsychina had particular directional feeding contingent on orientation of ovulate organs for access. For example, the tips of small *Williamsonia* organs likely were oriented in an upward manner whereas *Caytonia* integumental tubules were directed downwardly[21,84], allowing for different positions of the head and extended proboscis for efficient access to pollination drops.

Of the several, long-proboscid lineages of the Aneuretopsychina, Pseudopolycentropodidae is one of the more closely related lineages to Dualulidae. Various body features of Pseudopolycentropodidae, particularly mouthparts, inform interpretation of Dualulidae mouthpart structure and function, as both lineages exhibit similar structures. Pseudopolycentropodidae (Supplementary Fig. 7) and *Parapolycentropus* (Figs. 5a,b; 6; 7; Supplementary Figs. 8; 9a,b), have a proboscis that is formed from three separate, basic parts – two galeal halves that are joined to form a tube, and an elongate hypopharynx that evidently is variously modified to serve different mouthpart-related functions. Labral

and labial elements are well developed, providing bracing to the proboscis; mandibles are absent; and the maxillary region is often reduced. The proboscides in some taxa have dense, annular microtrichia and sclerotized bands on the outside surface of the galea; a maxillary palpus consisting of three articles, the third one is slightly longer than other two; and the absence of terminal absorptive structures such as pseudolabellae[49,50,69,77].

Five major comparisons exist for the mouthparts of Mesopsychidae, Aneuretopsychidae, Pseudopolycentropodidae, Dualulidae and *Parapolycentropus*, the latter formerly a member of the more phylogenetically distant Pseudopolycentropodidae but now closely related to the Dualulidae probably as a sister group (Fig. 1). First, Dualulidae, *Parapolycentropus* and Aneuretopsychidae have a distinctive external ornamentation and setal insertion patterns on their proboscides[39,49,50,69,83]. Mesopsychidae possessed randomly distributed, thick microtrichia and Pseudopolycentropodidae much less so, lacking recognizable surface structures as compression fossils[21,69]. Second, Aneuretopsychidae have a highly specialized and large, apparently fleshy, pseudolabellum that likely was a single structure[39,83]; by contrast, Mesopsychidae bore more diminutive pseudolabellae that were separated as two fleshy lobes on opposite sides of the proboscis terminus[21]. Pseudolabellae evidently do not occur among other mid-Mesozoic long-proboscid insects, although the proboscis in much older Permithonidae apparently had a modified (albeit incomplete) proboscis terminus with sclerotization and projecting setae[84]. Third, except in Aneuretopsychidae and Nedubroviidae that lack evidence for a hypopharynx, other long-proboscid groups clearly can be observed to have three parts – two conjoined galeal elements to form a tube and an intra-tubular hypopharynx. Additionally, Dualulidae and Pseudopolycentropodidae lacked the tight interlocking mechanism joining the galeal halves proposed for Mesopsychidae[21]. Most compression fossils of Pseudopolycentropodidae are preserved as sutured mouthpart elements evident along a section of the proboscis or are entirely disarticulated from the proboscis base (Supplementary Fig. 7). Fourth, although all long-proboscid taxa house a cibarial food pump under the clypeus, as well as an accompanying food tube, the much smaller salivary pump with a salivarium and salivary duct has only been securely established in Dualulidae (Supplementary Figs. 3a–e; 5b) and *Parapolycentropus* (Fig. 7d; Supplementary Figs. 8b–h; 9c–h). For functional reasons it is likely that Mesopsychidae and Aneuretopsychidae[20] had a salivary pump, but confirmation would require better preservation of additional specimens than exists at present[21,39,57,58,83,85]. Fifth, there is considerable variety in mouthpart movement and flexibility across the lineages. Some taxa can rotate or twist their proboscides almost 360°, such as Dualulidae and *Parapolycentropus*. Other taxa are significantly more limited in proboscis torsion and bending. Some had an ability to slightly bend their proboscides in a fixed direction, such as Mesopsychidae and most Pseudopolycentropodidae, whereas others taxa possessed a relatively stiff proboscis that disallowed any significant flexing, principally Aneuretopsychidae.

To more fully explore mouthpart microstructure, we took SEM scanning images of three specimens of *Pseudopolycentropus janeannae*[21], focusing on the base, midsection and terminus of each proboscis (Supplementary Fig. 7). At the base of the *P. janeannae* proboscis, the hypopharynx is largely covered by conjoined galeae; the hypopharyngeal salivary duct is inconspicuous. The maxillary palp is short, adpressed to the side of the labrum. At mid-proboscis, the hypopharynx is separated from the inner proboscis (galeal) surface, and it is at this point that sclerotized bands are clearly observed on the outer surface of the proboscis. This observation, especially in compression fossils, challenges previous statements that the only external structures on the proboscis were annular setae or microtrichia. At its terminus, the proboscis is split along the galeal suture, with only one galea is visible in a lateral, inner view, revealing the food tube inner surface that is very smooth compared to the uneven outside surface.

Compared to compression material, amber specimens display a greater number and more detailed features than compression specimens when observed under a stereoscopic microscope. For example, most specimens of amber *Parapolycentropus* reveal a delicate head and mouthpart microstructure, which can provide additional details when such features are viewed from different lighting angles. One

pair of dark, maxillary sclerites clearly can be seen in ventral view, consisting of a small, somewhat angulate cardo ridged in lateral view, which articulates with a longer and slender stipes that in turn connects with the palpifer (Supplementary Figs. 5b; 8a–c). The maxillary palp is three-segmented, occasionally is adjoined to the side of the labrum, but is physically separated from the proboscis (Figs. 3; 6d; 7h–j; Supplementary Fig. 8c–f,h). Although judging from its structure the palp is capable of considerable movement and flexibility, it is far too short for tactile contact of food at the proboscis terminus. However, some of these activities may be provided by an abbreviated maxillary palp with its three articles. The first article is short, thick, nearly a rectangular solid in shape, and principally serves as a connecting and supporting structure with the palpifer and stipes. The second maxillary article is fusiform and laden with several oval sensillae on the lateral surface, each including a macrotrichium in the center. The number and size of the various sensillae on the second maxillary article is uncertain, and may vary across specimens. The third, terminal maxillary article is substantially more slender than other two, and is densely covered with setae and sometimes upwardly oriented. Straddling the paired maxillary cardo and stipes sclerites on each side are the medially placed mentum of the labium, the most posterior of the head segment regions (Fig. 7j; Supplementary Figs. 5a,b; 8a–c). The mentum sclerite lies anatomically in the center of the ventral head region and often joins anteriorly the prementum. The prementum often is well developed, and it distinctly protrudes anteriorly where it adjoins the hypopharynx. The submentum is a triangular sclerite that is positioned posteriorly, is typically reduced in size and, sometimes is an inconspicuous, median sclerite. These labial sclerites – submentum, mentum and prementum – collectively appear elongate-rectangular in shape, are often vestigial, and are much narrower than the cardo and stipes of Pseudopolycentropodidae.

For Pseudopolycentropodidae, *Parapolycentropus* and *Dualula*, the clypeus and labrum have a smooth, external surface and house, respectively, the cibarial and salivary pumps below these sclerites. The cibarial pump, or food pump, provides suction through an expanding and contracting cibarial chamber for imbibition of incoming fluids through the proboscis food tube, and is substantially larger than the salivary pump. The salivary pump contains a chamber, the salivarium, which is attached to a valve (only preserved in *Dualula*) that allows the expelling of outgoing fluids through the narrow salivary duct at the proboscis terminus. These pumps likely are controlled by a series of compressor (cibarial pump) or circumferential muscles (salivary pump) that create negative and positive pressure, respectively, through the expansion and contraction of the cibarium and salivarium chambers visible in dorsal and lateral views (Fig. 3; Supplementary Figs. 8b–h; 9c–h; Supplementary Video 1). Between the inverted triangle of three ocelli above and the two antennal scapes below, a pair of frontal flanges (only observed in *Parapolycentropus*) are present on the head frontal surface (Figs. 6d; 7i; Supplementary Fig. 8b,d). Four or five types of frontal flanges are present in a variety of individuals in lateral view, consisting of columnar, semicircular, low-arched, oblique-triangular and irregularly shaped forms, but without an evident sex-based pattern. These unique structures could be the external expressions or the buttressing of internal apodemes for compressor muscle insertions to the cibarial pump. Connected to the cibarial pump are the long and flexible and conjoined galeae that form the food tube, consisting of an internally smooth surface, but with sclerotized, circumferential bands and annular setae on the external surface (Figs. 3; 6d,e,i; 7f; Supplementary Figs. 5a,b; 7; 8i–k,m,o). The food tube of conjoined galeae contains a hypopharynx that is slender, almost pellucid, and includes serrations on its center-ventral surface. Other than the serrations, the outer hypharyngeal surface is smooth, and lacks and setae, microtrichia, or a terminal acuminate or serrated structure for puncturing. It is notable that the hypopharynx preserves in its terminus, at the opening of the salivary duct, a small bubble with exuding fluids (Supplementary Fig. 8p, left). This indicates that there was outflow of fluids from the salivary duct, encouraged by the trauma of resin entombment. As well, there also are parallel exudations from the food tube resulting in much larger bubbles of egested fluid (Supplementary Figs. 8p–r).

Supplementary Note 3 │ *Dualula* Mouthpart Structure

The proboscis of *Dualula kachinensis*, monotypic member of Dualulidae (Figs. 1g–i; 3; Supplementary Figs. 3a–e; 5a,b), is a unique structure compared to closely related Pseudopolycentropodidae and *Parapolycentropus*[21,49,50,69,77], other, mid-Mesozoic, long-proboscid Mecoptera[21,49,50,84] (Supplementary Figs. 7; 8a–h; 9c–h), and remaining long-proboscid insect lineages past or present[84,86–90]. The two, major, active elements of the dualulid fluid-feeding apparatus are the modified hypopharynx with its supporting pump, and the surrounding maxillary galeae conjoined into an enveloping food tube that also houses its supporting pump.

The anatomically anteriormost elements, the clypeus and labrum, lack external ornamentation except for short peripheral setae and subtle labral ridges. The domed, polygonally shaped clypeus modified into a salivary pump. The maxillary elements of the head that provide the tubular galeae

---

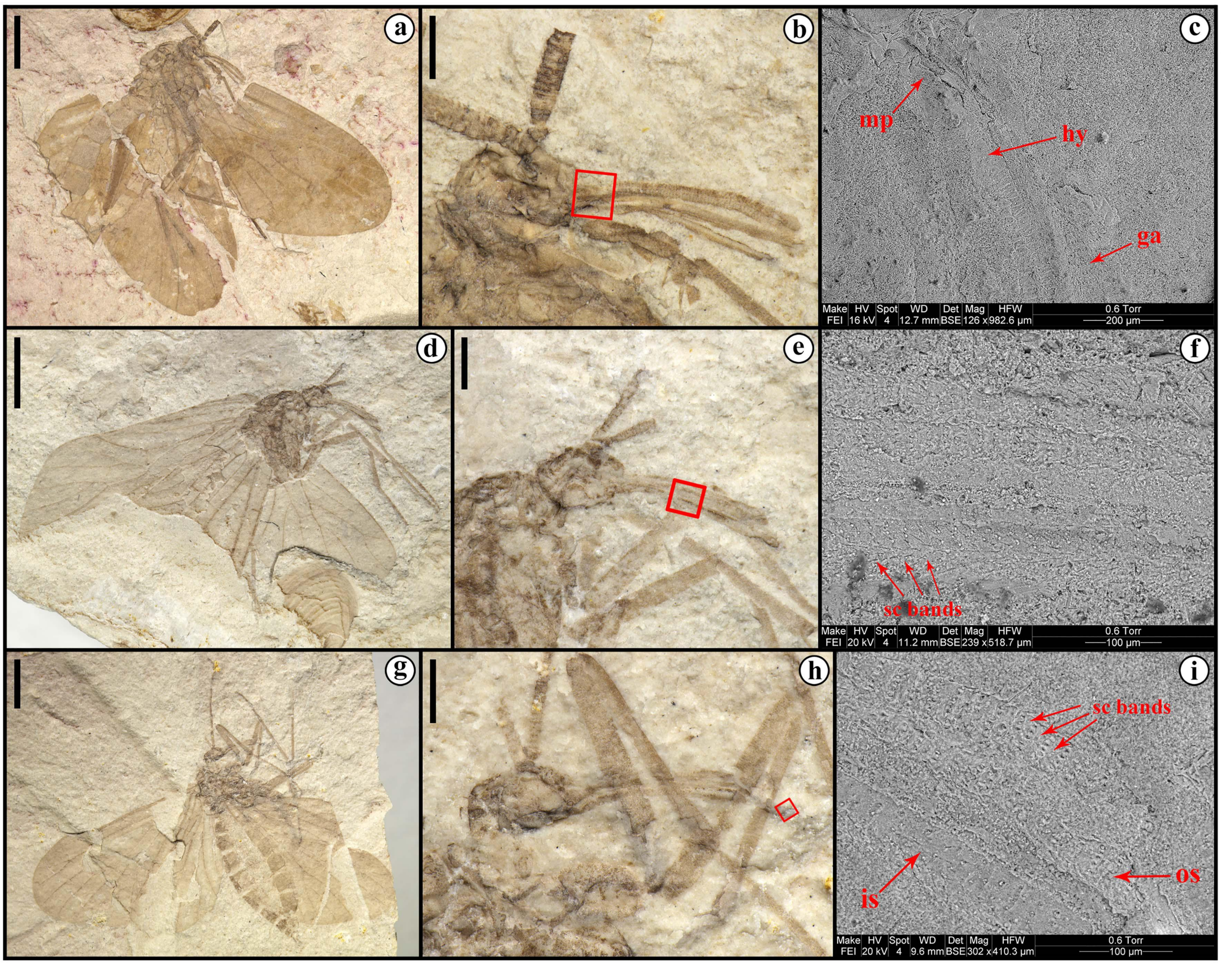

Supplementary Figure 7 │ Proboscis details of *Pseudopolycentropus janeannae*[21,49]. These specimens represent new material. (**a**), Specimen CNU-MEC-NN-2016008, sex unknown. (**b**), Specimen displaying thorax, head and proboscis, from (a). (**c**), SEM image enlarged from the template at proboscis base in (b). (**d**), Specimen CNU-MEC-NN-2016001P; sex unknown. (**e**), Enlargement of (d), showing thorax, head and proboscis. (**f**), SEM image enlarged from the template at mid proboscis in (d). (**g**), Specimen of CNU-MEC-NN-2016015P; female. (**h**), Enlargement of (g), showing thorax, head and proboscis. (**i**), SEM image enlarged from the template at proboscis tip in (h). Abbreviations: **ga**, galea; **hy**, hypopharynx; **is**, inner surface; **mp**, maxillary palp; **os**, outer surface; **sc bands**, sclerotized proboscis bands. Scale bars represent 2 mm in (a), (d) and (g); and 0.5 mm in (b), (e) and (h).

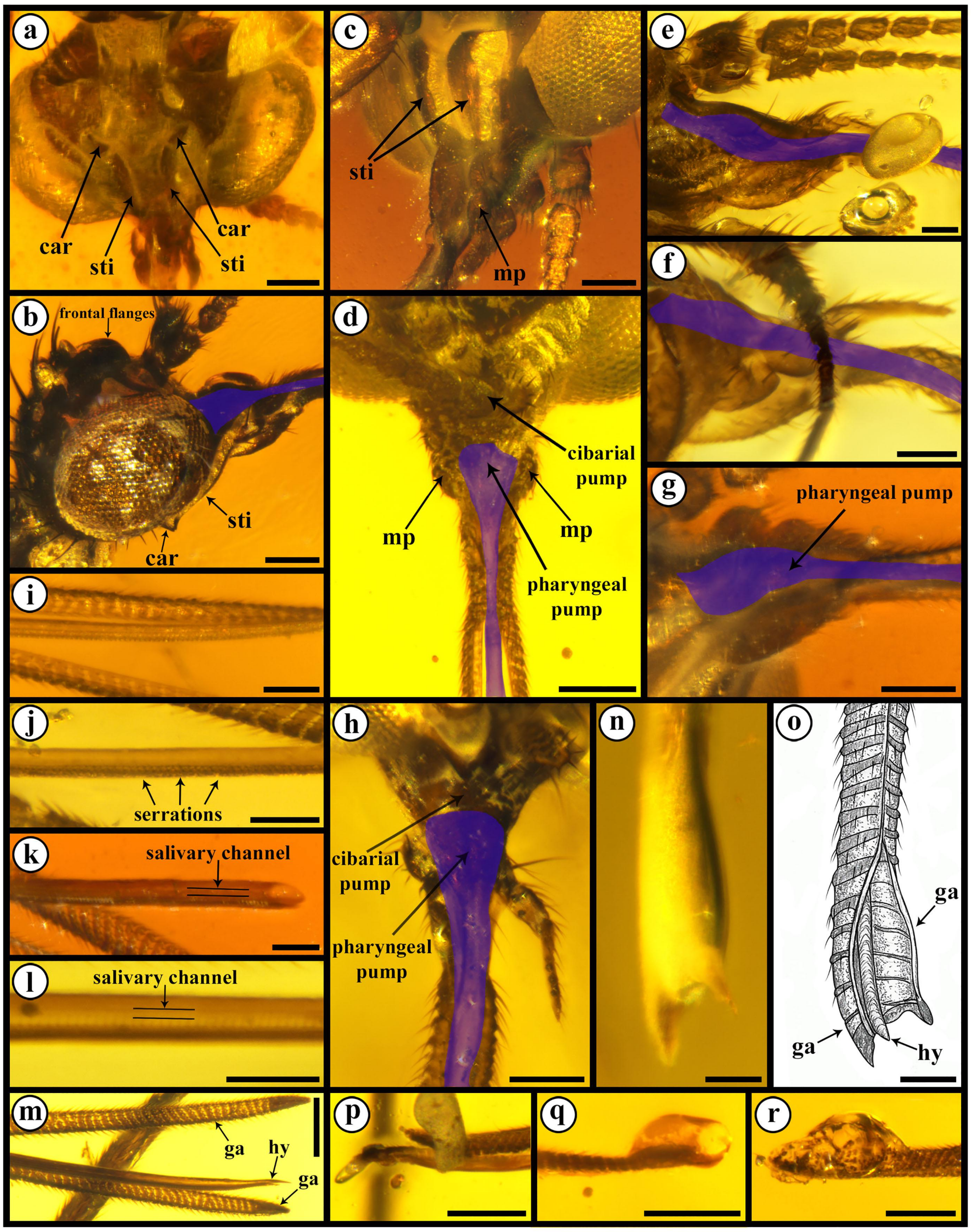

Supplementary Figure 8 │ Details of the proboscis base and other mouthpart elements of *Parapolycentropus* spp[50]. These specimens are new material. (**a**), Ventral view of head and mouthparts CNU-MEC-MA-2014005. (**b**), Right lateral view of head and mouthparts CNU-MEC-MA-2015052. (**c**), Ventral view of head and mouthparts CNU-MEC-MA-2015048. (**d**), Dorsal view of proboscis base CNU-MEC-MA-2015054. (**e**), Right lateral view of proboscis base CNU-MEC-MA-2017020. (**f**), Right lateral view of proboscis base CNU-MEC-MA-2015036. (**g**), Right lateral view of proboscis base CNU-MEC-MA-2015048. (**h**), Dorsal view of proboscis base CNU-MEC-MA-2014003. (**i**), Right lateral view of mid proboscis CNU-MEC-MA-2015055. (**j**), Left lateral view of hypopharynx CNU-MEC-MA-2017015, exhibiting serrations. (**k**), Right lateral view of hypopharynx CNU-MEC-MA-2015048, indicating the salivary duct. (**l**), Right lateral view of hypopharynx CNU-MEC-MA-2017007, with the salivary duct demarcated. (**m**), Right lateral view of proboscis terminus CNU-MEC-MA-2016016. (**n**), Dorsal view of proboscis terminus CNU-MEC-MA-2015037 in dorsal view, with its superimposed line drawing at (o). Lateral views of the proboscis termini CNU-MEC-MA-2017009 in (**p**), CNU-MEC-MA-2017013 in (**q**) and CNU-MEC-MA-2017014 in (**r**), showing extrusion of proboscis fluid during the resin entombing process. Purple denotes hypopharyngeal elements, including the basal, expanded, salivarium pump and the thin salivary duct to the terminus. Abbreviations: **car**, cardo; **ga**, galea; **hy**, hypopharynx; **mp**, maxillary palp; **sti**, stipes. Scale bars represent 0.1 mm in (a)–(d), (g), (h), (m) and (p)–(r); and 0.05 mm in (e), (f), (i)–(l), (n) and (o).

surrounding the salivary-pump complex originate from sclerites in the ventral part of the head capsule. These maxillary sclerites link to the proboscis base and occur lateral to the medially placed and often inconspicuous, elongate labial sclerite, the mentum. Each laterally placed set of maxillary sclerites consist of two elements: a small, angulate shaped cardo near the base of the head capsule and an articulating, relatively slender stipes that connects with the proboscis base and other maxillary elements (Supplementary Fig. 5a,b). The distal aspect of each stipes bears a lateral bulbous appendage, the palpifer, which supports the maxillary palpus, each composed of three compact articles and a terminal, filiform article that extends about two-thirds of the distance to the proboscis terminus. In some specimens, sensillae dot the surface of the second article. Another sex-based difference is the length of the third article in male Dualulidae (Supplementary Figs. 3a–d; 5a,b), which is about one-third of the length as the second article; by contrast, in females the same article is slightly longer than the second article. The highly reduced labium lacks palpi or palpifers, and occupies the narrow interstice between each lateral, articulating cardo–stipes pair. In some specimens, a triangular prementum sclerite is present and occasionally displays subtle features such as ridges.

The first and larger pump is the cibarial pump[91] that occurs under the clypeus and found in virtually all insect lineages regardless of mouthpart type. The cibarial pump in Dualulidae is evidenced by a bulge of the clypeus (Fig. 1i; Supplementary Fig. 5a,b), where it is supported on the surface by the clypeal sclerite (Fig. 3i; Supplementary Fig. 3d). The cibarial pump is powered by compressor musculature attached to an expandable chamber, the cibarium, for creation of suction via the esophagus for imbibition of fluid from the proboscis food tube[92]. In Dualulidae, the food tube of two sutured galeae is prominently ornamented by a series of sclerotized rings along each galeal half of the tube, and is diminished in intensity near the proboscis base and terminus. However, an alternative interpretation of the function of the conjoined galeal siphon recently has been proposed[69]. This earlier view, a misinterpretation in our view, posits the galeal siphon in Pseudopolycentropodidae as not a food tube, and rather serves a protective role by encompassing the salivary duct. Accordingly, the salivary duct was the proposed conduit for imbibition of fluid food[69]. Nevertheless, the salivary duct, because of its considerably narrower diameter than the food tube, would encounter resistance from inertial forces[93] that result from consumption of viscous food, disallowing passage of fluids that typically would flow freely in the galeal siphon of much wider diameter. Enzyme rich salivary fluids are considerably less viscous[94] and could be extruded through a salivary duct of much narrower diameter.

The second and smaller pump under the labrum is the salivary pump[91], a much smaller structure than the cibarial pump and is present in almost all piercing-and-sucking insects. The salivary pump in Dualulidae is a modification of the hypopharynx that consists of a hypopharyngeal base repurposed as a chamber, the salivarium, which serves as a contractile pump within the base of the galeal siphon. In *Parapolycentropus*, the well-exposed contractile salivarium (Supplementary Figs. 8b–h; 9c–h; Supplementary Video 1) contains salivary fluids whose outflow is regulated by a distally placed valve that is opened or closed with two, opposing dentate projections, as seen in *Dualula* (Fig. 3; Supplementary Fig. 3a–d). This condition is different from a valve with a single projection in extant aphids[95]. The salivarium valve controls outflow of salivary fluids along the tubular salivary duct of narrow diameter that extends to the distal proboscis terminus (Figs.1g,h; 3; Supplementary Figs. 3e; 5a,b), where it contacts fluid food on plant surfaces. This long salivary duct has a smooth outer surface, but displays regularly spaced serrations on along its ventral side, possibly serving as an attachment to the food tube inner surface. Notably, a salivarium valve was not detected in closely related *Parapolycentropus* or other Pseudopolycentropodidae, nor reported in other mid-Mesozoic scorpionflies. This absence, especially for compression specimens, may be attributable to insufficient preservation that would reveal microstructural features of the salivarium. Consequently, it is uncertain if other long-proboscid scorpionfly clades possessed a salivary pump

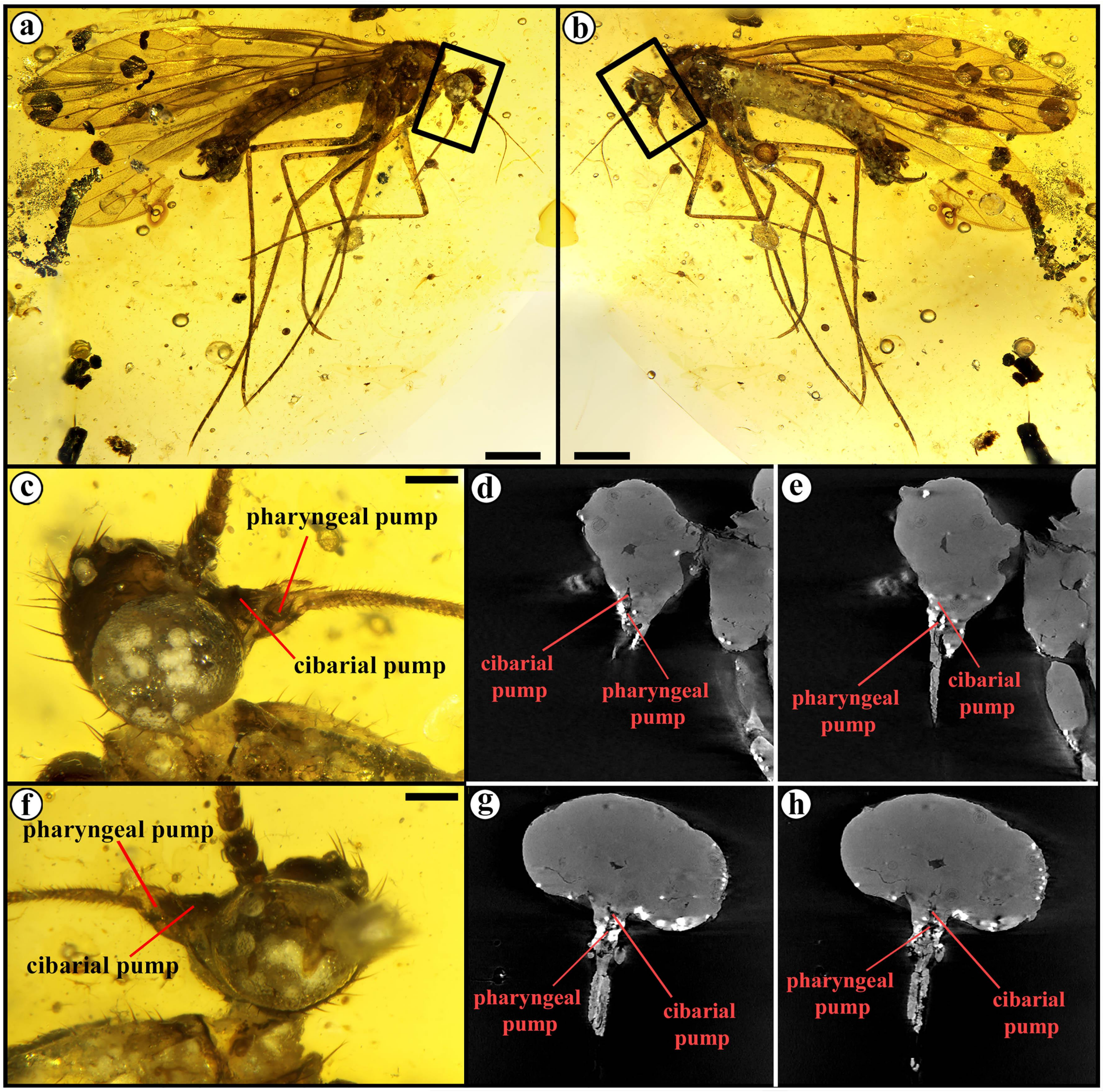

Supplementary Figure 9 │ Proboscis of *Parapolycentropus paraburmiticus*[50]. This figure includes Micro-CT images indicating cibarial (food) and salivarium (hypopharyngeal) mouthpart pumps from CNU-MEC-MA-2017008 (new material, male). (**a**)**,** Insect in right lateral view. (**b**), Insect in left lateral view. (**c**), Details of head and proboscis base in right lateral view. (**d**) and (**e**), Micro-CT image of the head and proboscis base in left lateral view, indicating the cibarial and salivary pumps in two sections. (**f**), Details of head and proboscis base in left lateral view. (**g**) and (**h**), The head and proboscis base in dorsal view, indicating the cibarial and salivary pumps in two sections. Scale bars represent 0.5 mm in (a) and (b), and 0.1 mm in (c) and (f). Images (d)–(h) are not to scale. For results of Micro-CT scanning, also see Supplementary Movie 1.

Supplementary Note 4 │ Feeding Processes and Food Sources of *Parapolycentropus* and Dualulidae

Mid Mesozoic, long-proboscid scorpionflies have been documented as probable pollinators of several extinct gymnosperm lineages and possibly early angiosperms. The evidence for biotic pollination has been overwhelmingly indirect, consisting of mouthpart structure[21,39,77,83] and features of microsporangiate organs, especially ovulate organs[21,94,96], consistent with access to rewards such as pollination drops, nectar and pollen[94]. Four major insect lineages of mid Mesozoic insects bear undisputed pollen grains and clumps adjacent insect bodies: Thysanoptera[97], Coleoptera[98,99], Neuroptera[86] and Diptera[89,96].

These lineages possessed highly specialized mouthpart structures and feeding habits[86,98], and were covered with pollen from one of the four groups of gymnosperms: Cycadales[99], Bennettitales[88], Pinales[86,96] and Ginkgoales[97]. Innovations such as small size, highly maneuverable wings, long-proboscate mouthparts and specialized feeding mechanisms enabled use of food sources associated with pollination[84,94]. A variety of evidence – mouthpart morphology, ovulate organ structure and insect associated pollen – are collectively important in understanding insect feeding habits, pollination and their co-associational relationships with plants.

Associations with Gymnosperms

The pollen grains mostly were distributed on the surface or adjacent the mouthparts and the middle and hind legs; fewer grains occurred next to the antennae; and the least number of grains were associated with the wings and forelegs (Fig. 6; Supplementary Fig. 10; Supplementary Data 3). For the same 54 pollen grains assessed, the average dimension is 12.15 μm for the long axis (range 9.69–15.21 μm) by 7.17 μm in equatorial diameter (range 5.46–9.24 μm), with a length to width ratio of about 1.70 (range 1.28–2.18) (Fig. 6c; Supplementary Data 3). The sulcus is elongate and almost the same length as the total length of the grain, and is slightly narrower in the middle, where the grain wall enrolls inwardly. The exine is opaque and psilate in ornamentation. The *Cycadopites* grains discussed here (Fig. 6) are substantially smaller than similar grains found on other Mesozoic insect bodies, such as five to eleven million-year-older Early Cretaceous Álava amber of Spain. Approximately 150 Álava amber *Cycadopites* grains of probable ginkgoalean affinity were described attached to and adjacent the bodies of *Gymnopollisthrips*, a melanthripid thrips[97]. These attached and associated grains had average dimensions of 20.4 μm in length (range 17.4–24.9 μm) by 12.6 in equatorial diameter (range 9.3–15.4 μm)[97]. By contrast, the probable cycad affiliated *Monosulcites* pollen on the oedemerid beetle *Darwinylus* had average dimensions of 25.14 μm in length (range 38.57–18.85 μm, N=62) by 16.56 μm in equatorial diameter (range 11.22–28.11 μm, N=69)[98]. This pollen type on *Darwinylus* possessed a rather open, granular sulcus suggesting cycad affinity. The relevant affiliation accommodated by the 12.15 μm average length of our *Cycadopites* sp. pollen grains (Fig. 6; Supplementary Fig. 10; Supplementary Data 3) remains unknown, although some bennettitalean pollen grains approach these small pollen grains in size[100]. Very few described *Cycadopites* taxa approach this size; the closest is a dispersed pollen of unknown affinities from the Late Cretaceous of Austria with a diameter about 12–15 μm in long axis[101]. Notably, *Cycadopites* pollen has not been found to occur in any angiosperm flower. In addition, small, bowl-shaped, neutral hued, nonshowy angiosperm flowers like *Amborella*[96,99] antedate by a few tens of millions of years[98] the earliest occurrence of tubular, deep-throated flowers consistent with long-proboscid pollination[102,103].

Insect pollinators of *Tropidogyne* and other cup-shaped flowers, based on their floral structure and evidence from pollination modes of modern basal angiosperms[96,103], would have been small beetles, midges, small nematocerous flies, thrips, parasitoid wasps and early moths[96,102,103]. Large-bodied insects with considerably larger long-proboscides (Supplementary Data 4) could not interact for a lack of fit. Pollinators with appropriate proboscides would include small, mosquito-sized pseudopolycentropodid, *Parapolycentropus* and dualulid scorpionflies that functionally would have been similar to early moth pollinators. Notably, angiosperm nectar would have been an uncommon resource given the rarity of floral nectaries on basal angiosperm lineages[99,102], and may have replaced pollination-drop consumption as a reward in many insect groups[94]. Although the two *Parapolycentropus* specimens described here are associated with two different types of pollen grains, gymnosperm and, possibly, angiosperm, these disparate associations may record the transition from a gymnosperm to angiosperm plant hosts during the Aptian–Albian Gap[98,103]. Such a transition would be similar to that of Oedemeridae, a lineage of beetles that included Early Cretaceous cycad or bennettitalean as well as modern angiosperm pollinators[98]. The difference, however, between the two examples is that Oedemeridae successfully

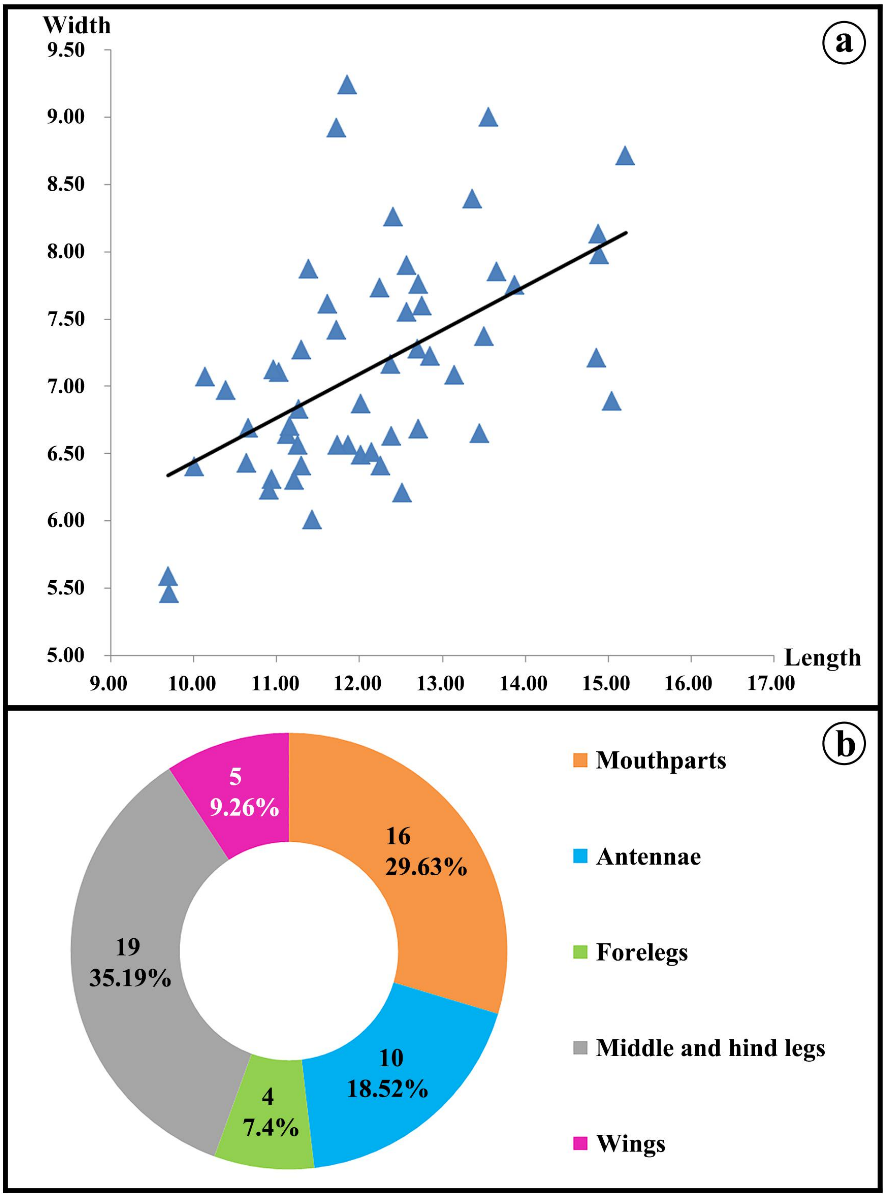

Supplementary Figure 10 │Measurements and distribution of *Cycadopites* sp. pollen grains adjacent to *Parapolycentropus paraburmiticus*. CNU-MEC-MA2017012, new material, male. (**a**), Scatter plots of pollen grain length and width, and there is a trend line to denote the changes in pollen size. Horizontal axis is the length of pollen, and vertical axis is width, measurements in micrometer (μm). (**b**), Doughnut chart shows the distribution of pollen grains associated with the insect body, the numbers on different color blocks are the specific number (above) and percentage (below) of the pollen grains.

---

transitioned and is extant, whereas the fossil record indicates that *Parapolycentropus* soon became extinct after a possible host-plant switch[98,103,104].

Possible Associations with Angiosperms

The presence of structurally distinctive *Cycadopites* pollen (Fig. 6) and five morphotypes of early angiosperm flowers (Supplementary Fig. 11) in Myanmar Amber are notable for their diminutive size compared to other, similar plant structures that typically are considerably larger in floral assemblages from other mid-Mesozoic intervals[97,105]. The micropyles of large seeds, often with unknown plant affinities, and ovulate reproductive structures containing tubes, channels, catchment funnels and pappus

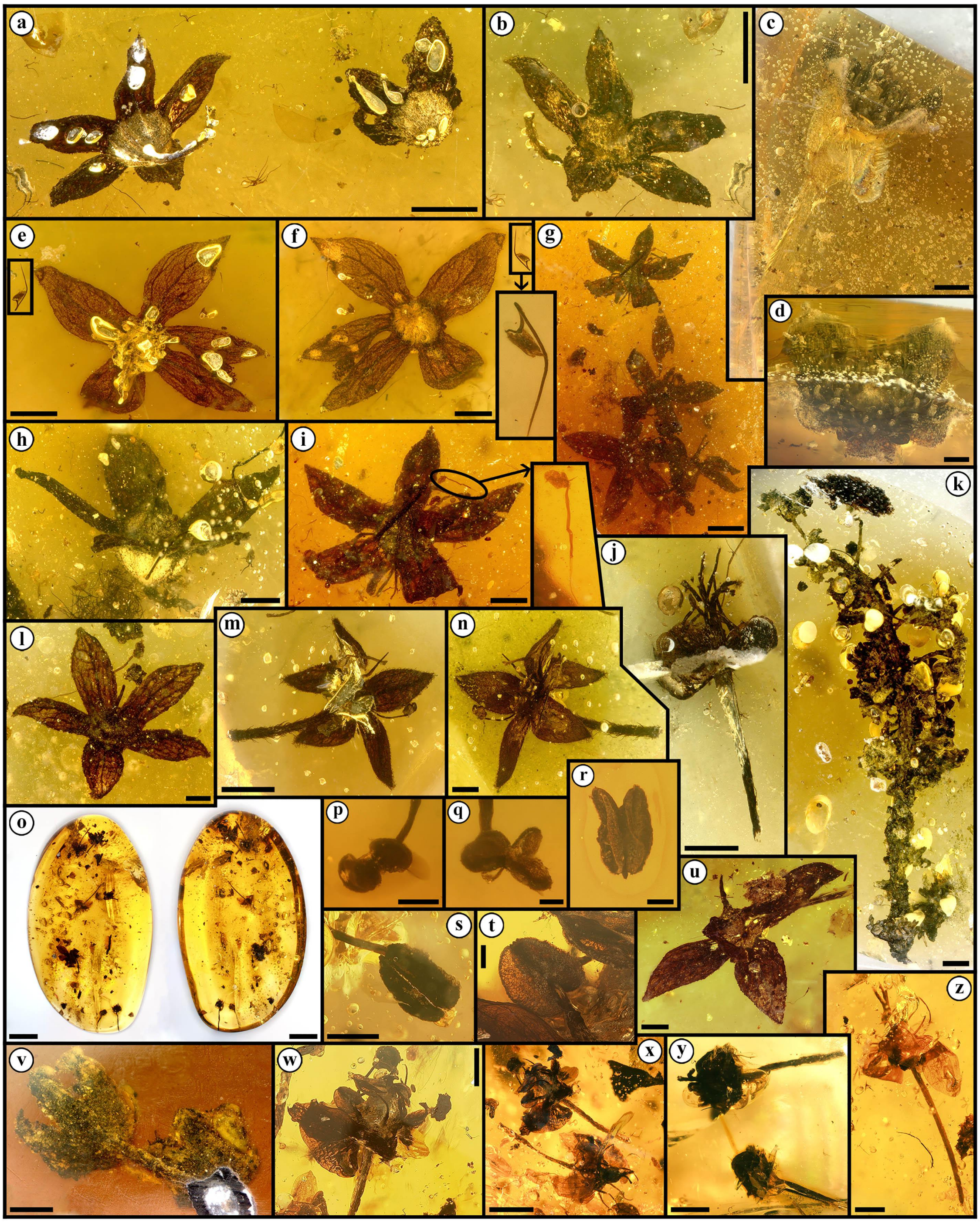

Supplementary Figure 11 │ *Tropidogyne* spp. and five angiosperm flower morphotypes with attached stamens from Myanmar amber. (**a**), Flower CNU-PLA-MA-2015018 in dorsal view, left (2015018-1) is *Tropidogyne pikei* and right (2015018-2) is *Tropidogyne pentaptera*. (**b**), Flower CNU-PLA-MA-2015018-1 in polar view. (**c**), Flower CNU-PLA-MA-2015022 in lateral view, Morphotype A. (**d**), Flower CNU-PLA-MA-2015022 in polar view. (**e**), Flower CNU-PLA-MA-2015023 in polar view. (**f**), Flower CNU-PLA-MA-2015023 in dorsal view with enlargement of one stamen. (**g**), Flower CNU-PLA-MA-2015023 with four flowers in one amber piece, and enlargement of one flower in (**i**) with a clear stamen. (**h**), Flower CNU-PLA-MA-2015024 in lateral view. (**j**), Flower PAL 631404 in lateral view, Morphotype B. (**k**), Flower CNU-PLA-MA-2015047, a partly preserved generative shoot, Morphotype C. (**l**), Flower CNU-PLA-MA-2015026 in polar view. (**m**) and (**n**), Flower CNU-PLA-MA-2015029 in lateral view with two anthers in (**p**) and (**q**). (**o**), Several flowers from CNU-PLA-MA-2016001. (**u**), Flower CNU-PLA-MA-2017001 in lateral view. (**v**), Flower CNU-PLA-MA-2015028 in lateral view, Morphotype D. (**w**) to (**z**), Several flowers in CNU-PLA-MA-2016001 with three anthers illustrated in (**r**) to (**t**), all Morphotype E. Floral details are indicated in Supplementary Data 7. Scale bars represent 1 mm in (a)–(c), (g), (j), (k), (m), (w), (y) and (z); 0.5 mm in (d)–(f), (h), (i), (l), (n), (s), (u) and (v); 0.2 mm in (p) and (t), (r) and (t); 0.1 mm in (q); 2 mm in (x); and 5 mm in (o).

tubes[21,84] from which pollination drops were secreted are candidates for long-proboscid extraction of fluids by small-bodied insects such as Pseudopolycentropodidae, *Parapolycentropus* and Dualulidae. A seemingly unlikely plant host would be rather small angiosperm flowers such as *Tropidogyne*[106,107] (Supplementary Fig. 11a,b,e–i,l–n,u), particularly as they lack a distinct tubular corolla. However, their cup-shaped form could have accommodated small, mosquito-size insects with long-proboscid aspect ratios, such as small moths, *Parapolycentropus* and *Dualula*. Based on their morphologies, these flowers likely secreted stigmatic fluids and perhaps bore floral nectaries to attract insects such as small beetles, thrips, parasitoid wasps, short-proboscid glossate moths, midges, labellate flies[94,96,101,108,109] and small scorpionflies. *Parapolycentropus* could have pollinated Morphotype E, a cup-shaped flower represented by four specimens (Supplementary Fig. 11r–t, w–z; Supplementary Data 6). Flower Morphotype A exhibits an average sepal length of 1.78 mm, although the distance from the sepal tip to the gynoecium would be somewhat shorter. The longest measured proboscis lengths of *P. burmiticus* is 1.53 mm (N=22) and *P. parapolycentropus* is1.50 mm (N=51), with no evident difference in male versus female proboscis lengths (Supplementary Data 4). These floral depths and proboscis lengths, taking into account a 0.9 mm difference resulting from the average sepal lengths minus the longest proboscis lengths, would allow elevation of the gynoecium above the floor of the corolla base, indicating an accommodating match (Supplementary Data 5 and 6). Additional pollinators may have associated with the more bowl-shaped flowers of *Tropidogyne pentaptera* with an average floral depth of 1.97 mm (N=5), and *T. pikei*, with a depth of 1.89 mm (N=3). We note that a possible angiosperm pollen grain adjacent a *Parapolycentropus* proboscis (Fig. 7e), suggests the presence of insect pollination.

Dualula kachinensis has a proboscis length that is considerably smaller and shorter than compression mesopsychid, aneuretopsychid and pseudopolycentropodid taxa (Supplementary Data 4), but larger and longer than both species of *Parapolycentropus* mentioned above (Supplementary Data 5). Considering that the single, measured complete proboscis of *D. kachinensis* is 3.23 mm, it would appear that none of the floral morphotypes – including Morphotype A, *T. pentaptera* and *T. pikei* – was sufficiently deep to accommodate the *D. kachinensis* proboscis (Supplementary Data 5 and6). The absence of an adequate fit indicates that *D. kachinensis* accessed pollination drops of other, probably gymnosperm, ovulate organs or possibly flowers with corollas approximately 1 mm deeper. Suspect candidates for access by *D. kachinensis* are *Samaropsis* ovules (micropyles), *Caytonia* fructifications (integumental tubes) and *Alvinia* cones (catchment funnels)[84,94,110–112], that occur in earlier compression deposits but may have had morphological analogs in Myanmar Amber. For *D. kachinensis*, the likely food was the nutritive liquid of gymnosperms and probably small, early angiosperm flowers with associations similar to small, modern moths on a variety of small angiosperm flowers[113]. Notably, the occurrence of Myanmar Amber with small, long-proboscid scorpionflies and early glossate moths, is at the end of the Aptian–Albian Gap, in which the shift from a gymnosperm to an angiosperm dominated global flora was well under way[98].

Supplementary Note 5 | Hind-Wing Reduction in Mid-Mesozoic Insects, Pseudopolycentropodidae, *Parapolycentropus* and Dualulidae

Drosophila melanogaster unquestionably has been the preeminent model organism for studies in evolutionary developmental biology for nearly the past 60 years[60,114]. During this time, regulatory mechanisms by homeotic containing genes (Hox genes) were known to play an important role the development of embryos. Simultaneously, there were studies of model insects, including those other than *D. melanogaster* that indicated features such as segmental identity and structures anatomically linked to particular segments, such as wings[115–119], were produced by the action of Hox genes on particular target sites of the organism[116]. Such Hox genes were highly conserved under normal conditions and typically had three primary consequences after their activation. One result was the

change from one original structure to another, for example the transformation of a wing to a haltere or a leg. The other two functions of Hox genes were to suppress expression and detect and regulate structural change[117].

It was the first action, that two externally different but serially homologous structures could be changed into each other's phenotype, that became relevant for understanding how more subtler transformations, such as the change of a wing to a haltere would be developmentally possible. Among Hox-controlled genes, the *Ultrabithorax* (*Ubx*) gene could implement such a transformation of wings into halteres, and in the process suppress the development of a normal, membranous wing in *D. melanogaster*. Specifically, if the function of *Ubx* were removed in the metathoracic (T3) segment, halteres would develop into rather normal, membranous, fully veined wings[115,118]. Consequently, under blocked *Ubx* function and absent any intervening genetic changes, the transformation of wings to halteres is not attainable. In 2011, Pavlopoulos and Akam[118] designed experiments that would further address the morphological and molecular transformations after the inactivation of *Ubx* by induced temperature shift from 19℃ to 29℃. Their results revealed that ambient temperature is major factor that influences overall *Ubx* expression, and these changes not only happened in organ development but also at the cell level[118]. Additionally, this modulation by the *Ubx* gene has proved to target a particular developmental stage of the *D. melanogaster* embryo, and the switch from a normal wing to a haltere involves specification of other target genes in a complicated regulatory process.

Meanwhile, previous studies of insect flight biomechanics had indicated that dipterans were among the most agile of flying animals. This is a conclusion often attributed to typical functions of the modified hind wings (halteres) of Diptera. Early research by Pringle initially elucidated the flight dynamical mechanisms of the halteres in higher dipterans[119]. Thereafter, subsequent experiments using a variety of methods documented the role that halteres played in ensuring flight stability, agility and endurance by their function as gyroscopic sensors that provided neurosensory feedbacks to the forewings[120,121]. In pollination of flowers[84,105], gyroscopic control of flight suggests that a hovering position over gymnosperm reproductive organs occurred as they imbibed pollination drop fluids[89,94]. In addition, dipterans likely exhibited rapid and accurately relayed neural responses for high-speed body movements and large-angle, swerving manoevers[122,123], resulting in energetically efficient behaviors of plant foraging and evasion of predators. Owing to the haltere-like hind wings and small body size, early mecopterans would obtain advantages; for instance, greater sensitivity to airflow agitation in favor of making a rapid turns when eluding predators. During the mid Mesozoic, such advantages also became increasingly beneficial to larger, long-proboscid dipterans, such as rapidly hovering tanglevein flies (Nemestrinidae)[84]. The highly flickering wingbeat of tanglevein flies is indicated in their wings by anteriorly directed longitudinal veins[123] and their current pollinator interactions with deep-throated flowers.

Unfortunately, data from embryonic developmental experiments are lacking that would provide definitive evidence in extant mecopterans to support the conclusions from a variety of wing modifications we found in mid-Mesozoic Pseudopolycentropodidae, *Parapolycentropus* and *Dualula*. We hypothesize that these mid-Mesozoic long-proboscid lineages shared the same evolutionary developmental genetic mechanisms as modern *Drosophila*[116] and indeed all Diptera. It would be illuminating for future experimental studies to understand how these Hox-controlled genes regulate the formation of novel structures in modern Mecoptera[115]. Such explorations would provide a new evolutionary developmental perspective for understanding the change from a four-winged to a two-winged mecopteran.

Supplementary Note 6 │ Genitalia structure in Mesopsychidae, Pseudopolycentropodidae, *Parapolycentropus* and Dualulidae

The male genitalia of Dualulidae (Supplementary Fig. 5c–e) and *Parapolycentropus*[69] (Figs. 6a,b; 7a,b; Supplementary Fig. 9a,b) are unique and significantly different from the male genitalia of most mecopterans (Supplementary Figs. 13,14) and all dipterans. A peculiarly distinctive feature is the upturned gonostylus (Supplementary Figs. 5c–e; 11g,h; and Fig. 8 in [69]).The male genitalia of extant

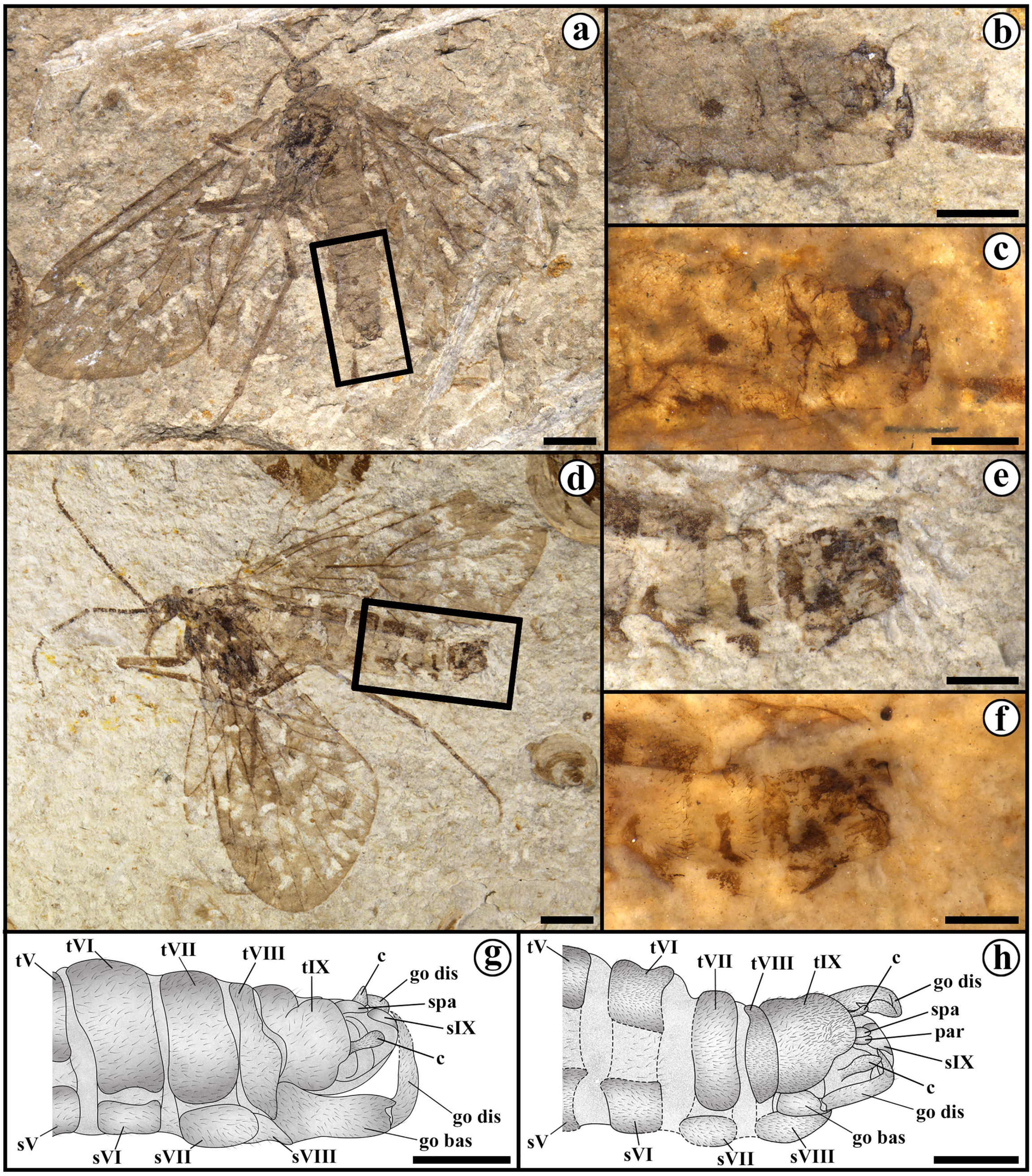

Supplementary Figure 12 │ Male genitalia of *Pseudopolycentropus janeannae*[21,49]. Examined specimens are CNU-MEC-NN-2017050 (new material), and CUN-MEC-NN-2005004C, a paratype[21]. (**a**), Specimen of CNU-MEC-NN-2017050. (**b**), Male genitalia of (a) from template in (a). (**c**), Male genitalia of (b) under alcohol. (**d**), Specimen CNU-MEC-NN-2005004C. (**e**), Male genitalia from template in (d). (**f**), Male genitalia of (e) under alcohol. (**g**), Line drawing of the male genitalia from (b) and (c). (**h**), Line drawing of male genitalia from (e) and (f). Scale bars represent 1 mm in (a) and (d); 0.5 mm in (b), (c) and (e)–(h).

scorpionflies appear considerably different, and the most specialized groups such as Panorpidae[52,125], bear male genitalia that are enlarged and uplifted above the scorpionflies' dorsum, akin to the position of a scorpion metasoma and sting. However, for extinct taxa, most lineages lack well-preserved body structures. For instance, in the long-proboscid clade of Aneuretopsychina, Liassophilidae and Permotanyderidae[26,47,85,126,127], are the sister group to a Dualulidae + *Parapolycentropus* clade in the resulting trees (Fig. 1; Supplementary Fig. 1i). However, Liassophilidae and Permotanyderidae only include fossils preserved as compressions. Most constituent species are classified entirely on venational features[26,34,47,125–127]. In Aneuretopsychina, except Permian Nedubroviidae, the other three families have partly preserved abdominal features[21,39,56,57,83,87], but these records lack informative descriptions of genitalia.

We have reviewed all described specimens of Aneuretopsychina, evaluated relevant published papers and 37 new fossils of Mesopsychidae (15 specimens) and Pseudopolycentropodidae (22 specimens) from the CNU paleontological collections. We found four, clearly preserved, compression–impression fossils that bore relatively complete male genitalia. These new fossils were collected from Daohugou Village, Ningcheng County, of Inner Mongolia in Northeastern China. The fossils originate from the Jiulongshan Formation and are of latest Middle Jurassic age at 164 Ma[128,129]. From camera lucida

---

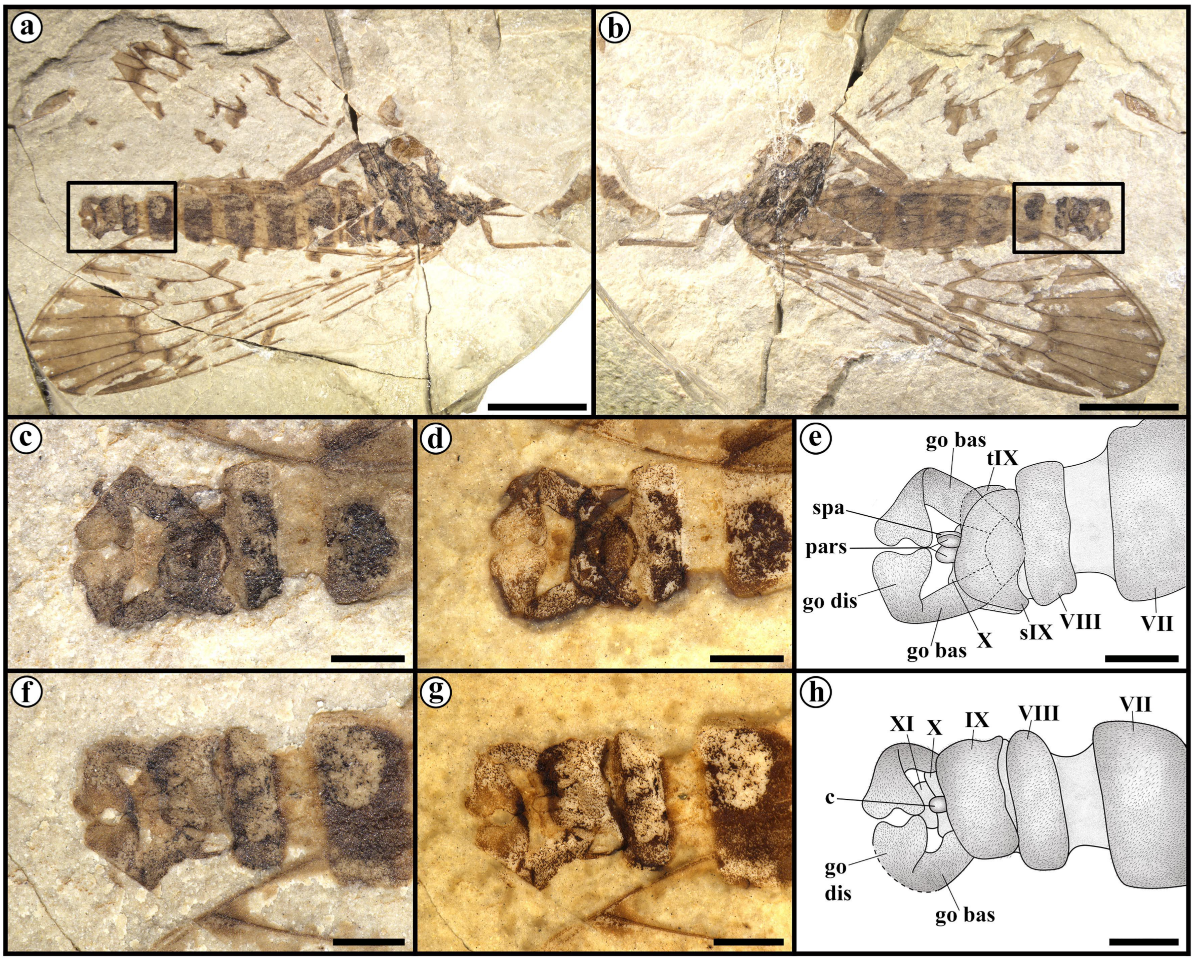

Supplementary Figure 13 │ Male genitalia of *Lichnomesopsyche daohugouensis*[21,57]. The specimen is CNU-MEC-NN-2016019P/C, new material. (**a**), Specimen part. (**b**), Specimen counterpart. (**c**), Male genitalia from template in (a). (**d**), Male genitalia of (c) under alcohol. (**e**), Line drawing of male genitalia in (c) and (d). (**f**), Male genitalia outlined by template in (b). (**g**), Male genitalia of (f) under alcohol. (**h**), Line drawing of male genitalia from counterpart. Scale bars represent 5 mm in (a) and (b), and 1 mm in (c)–(h).

drawings and subsequent comparisons of the genitalic features of these specimens with other species of Aneuretopsychina, we found the most important similarities were claspers, each of which consisted of a basistylus (or gonocoxa) and a dististylus. In Mesopsychidae, the gonocoxa presents very thick and stout structures, especially for *Lichnomesopsyche daohugouensis*[21,57] (Supplementary Fig. 13). This species has a concavity on the tip of each dististylus, probably deployed for grasping a conspecific female abdomen during copulation. Another species of Mesopsychidae, *Epicharmesopsyche pentavenulosa*[87], bore claspers that were much longer and robust, but with a somewhat smaller concavity on the cusp of both dististyli (Supplementary Fig. 14). The male genitalia of *Parapolycentropus* in amber bore considerable general similarity to Pseudopolycentropodidae in compression fossils[49, 69] (Supplementary Fig. 12). The structures of these two last-mentioned taxa are quite similar, particularly the upwardly projecting dististylus and enlarged tIX tergum (Supplementary Fig. 14). An additional similarity is the presence of two ameristic cerci, one superanale and two paraprocts, exhibiting minimal differences in size and shape. Unrelated features, by comparison to other records of pseudopolycentropodids, are the two-winged mecopterans that have reduced sternites sII to sVI, indicating a structure associated with feeding on nutritious liquids[69]. In general, there were considerable, likely homologous, similarities among the male genitalia of Mesopsychidae, Pseudopolycentropodidae (including *Parapolycentropus*) and *Dualula*.

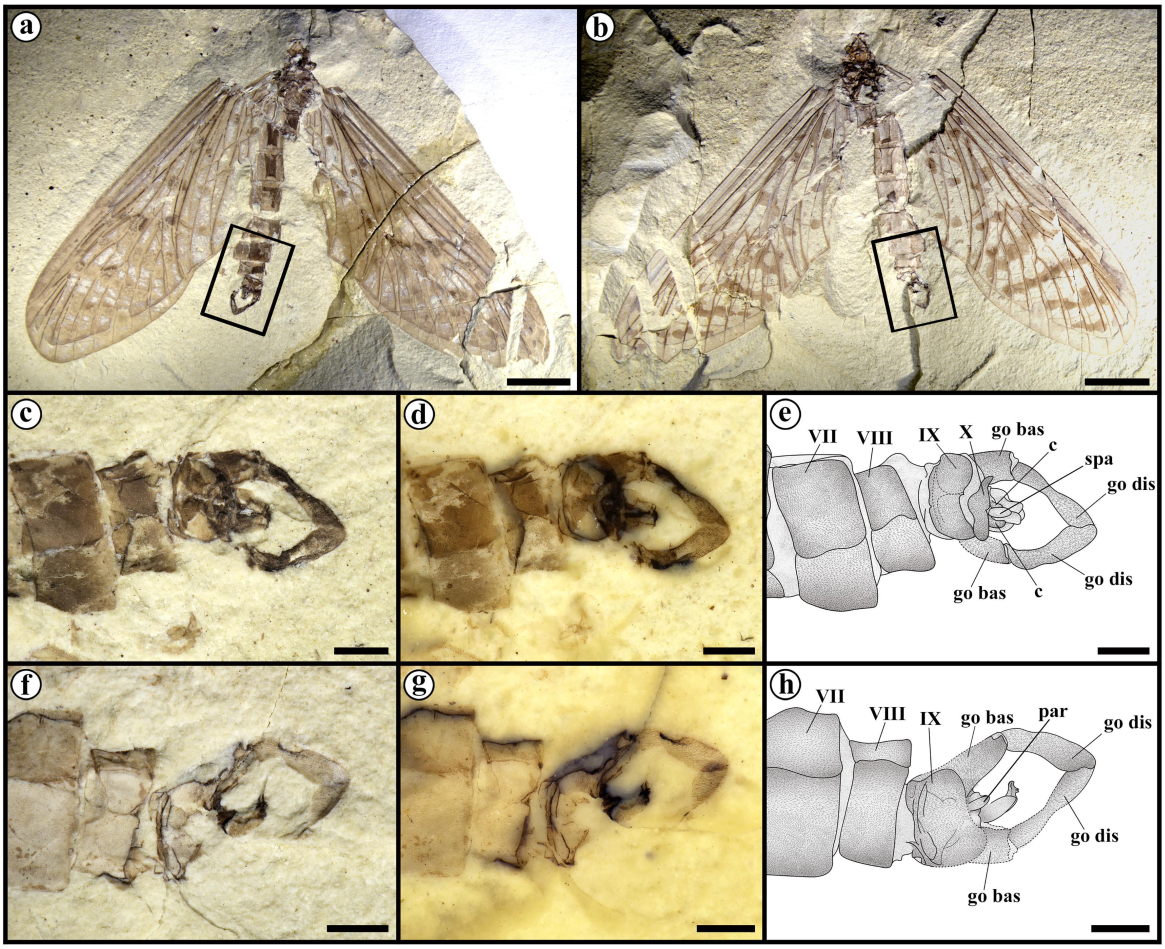

Supplementary Figure 14 │ Male genitalia of *Epicharmesopsyche pentavenulosa*[87]. The specimen is CNU-MEC-NN-2015005P/C, new material. (**a**), Specimen part. (**b**), Specimen counterpart; **c**, Male genitalia outlined by template in (a). (**d**), Male genitalia of (c) under alcohol. (**e**), Line drawing of male genitalia in (c) and (d). (**f**), Male genitalia outlined by template in (b). (**g**), Male genitalia of (f) under alcohol. (**h**), Line drawing of male genitalia in (f) and (g). Scale bars represent 5 mm in (a) and (b), 1 mm in (c)–(h).

Supplementary Note 7 | Reproductive Biology of *Parapolycentropus* and Dualulidae

Many insects engage in swarming behavior that is beneficial for congregation, mating and dispersal, particularly among Holometabola such as Hymenoptera, Lepidoptera, Trichoptera and Diptera[130]. The fossil record contains examples of swarming behavior, especially the social insects of termites, ants and bees[131–133], indicating that such behavior embodies a highly coordinated assembly of individuals gathering in a circumscribed site. The majority of fossil insect swarms consist of Diptera preserved in a variety of

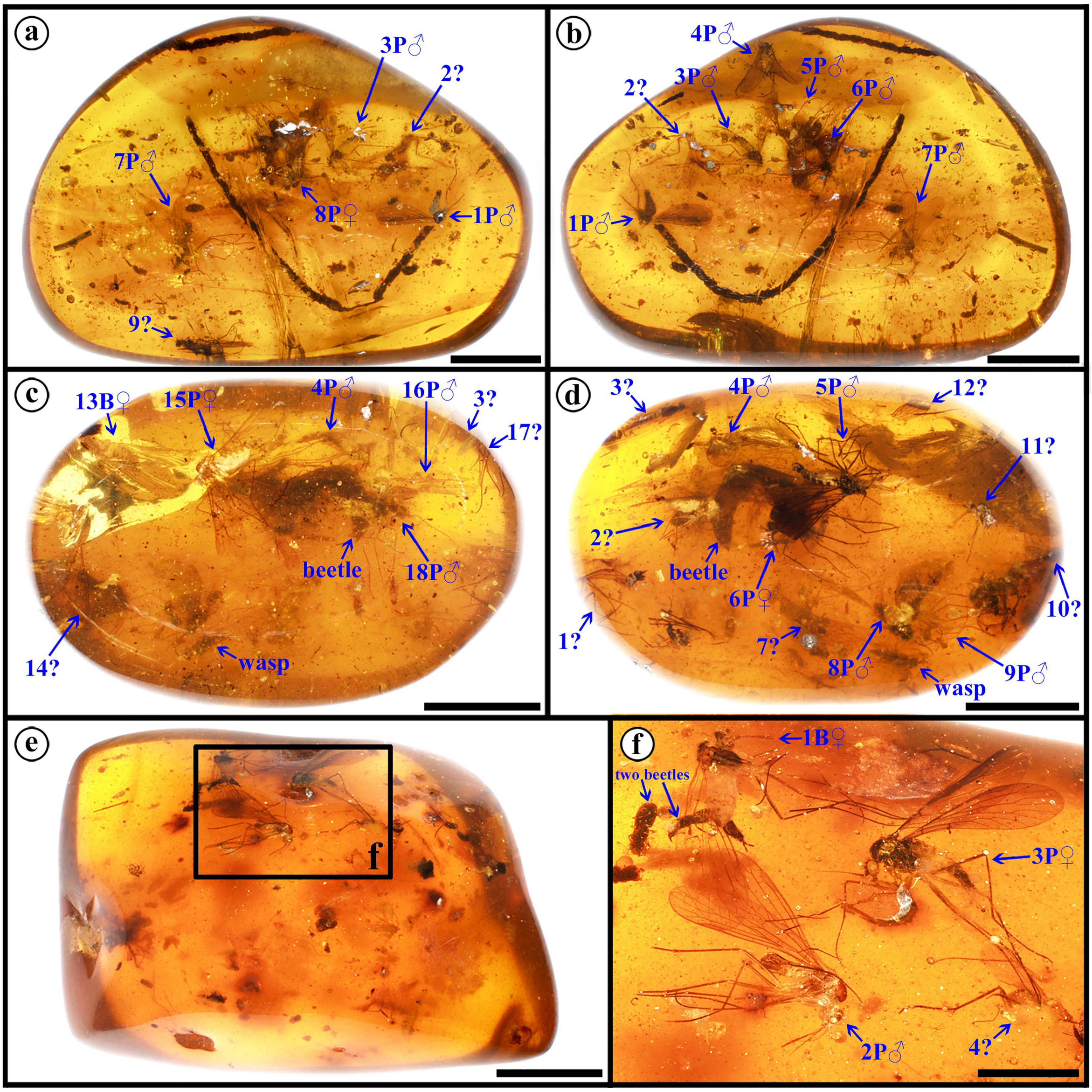

Supplementary Figure 15 | Three entombed swarms of *Parapolycentropus* spp.[50,69] (**a**), Reverse view of six visible individuals in specimen CNU-MEC-MA-2016007. (**b**), Obverse view of seven visible individuals in (a) among nine total insects. (**c**), Reverse view of eight visible individuals in CNU-MEC-MA-2015038. (**d**), Obverse view of individuals in (c), with 12 individuals visible among 18 total *Parapolycentropus* individuals in the surrounding amber, including a beetle and a wasp. (**e**), Specimen CNU-MEC-MA-2015030. (**f**), Obverse view of individuals in (e), exhibiting the clustering of four *Parapolycentropus* individuals and two beetles. Abbreviations: **B**, *Parapolycentropus burmiticus*; **P**, *Parapolycentropus paraburmiticus*; and the question marks denote *Parapolycentropus* sp. and sex unknown. Scale bars represent 5 mm in (a), (b) and (e); 4 mm in (c) and (d); and 2 mm in (f).

ambers, with provenances from youngest to oldest, in the Dominican Republic, Baltic Region and associated Rovno area in Ukraine, Canada, France, Myanmar, Spain and Lebanon[131,132,134–136]. Modern groups of nematocerous Diptera, for example, Culicidae, Chironomidae and Ceratopogonidae; brachycerous Diptera including Bombyliidae; Tabanidae; Empididae and a few Cyclorrhapha such as Phoridae have been observed in mating flights[137]. One type of swarming reproductive behavior in dipterans is lekking, the participation in an airborne communal mating event[137]. However, this phenomenon rarely has been reported in extinct or extant mecopteran species, attributable, at least in modern groups, to scorpionflies requiring high levels of ambient food, or possessing a relatively narrow geographical distribution, or characterized by small population levels when compared to other Holometabola. Based on the imperfect preservational condition of most compression fossils, mecopteran lek events would be difficult to detect in the fossil record. Although lek events rarely are documented in the fossil record, the Nannochoristidae from the Middle Jurassic of Northeastern China[112] evidently is an exception.

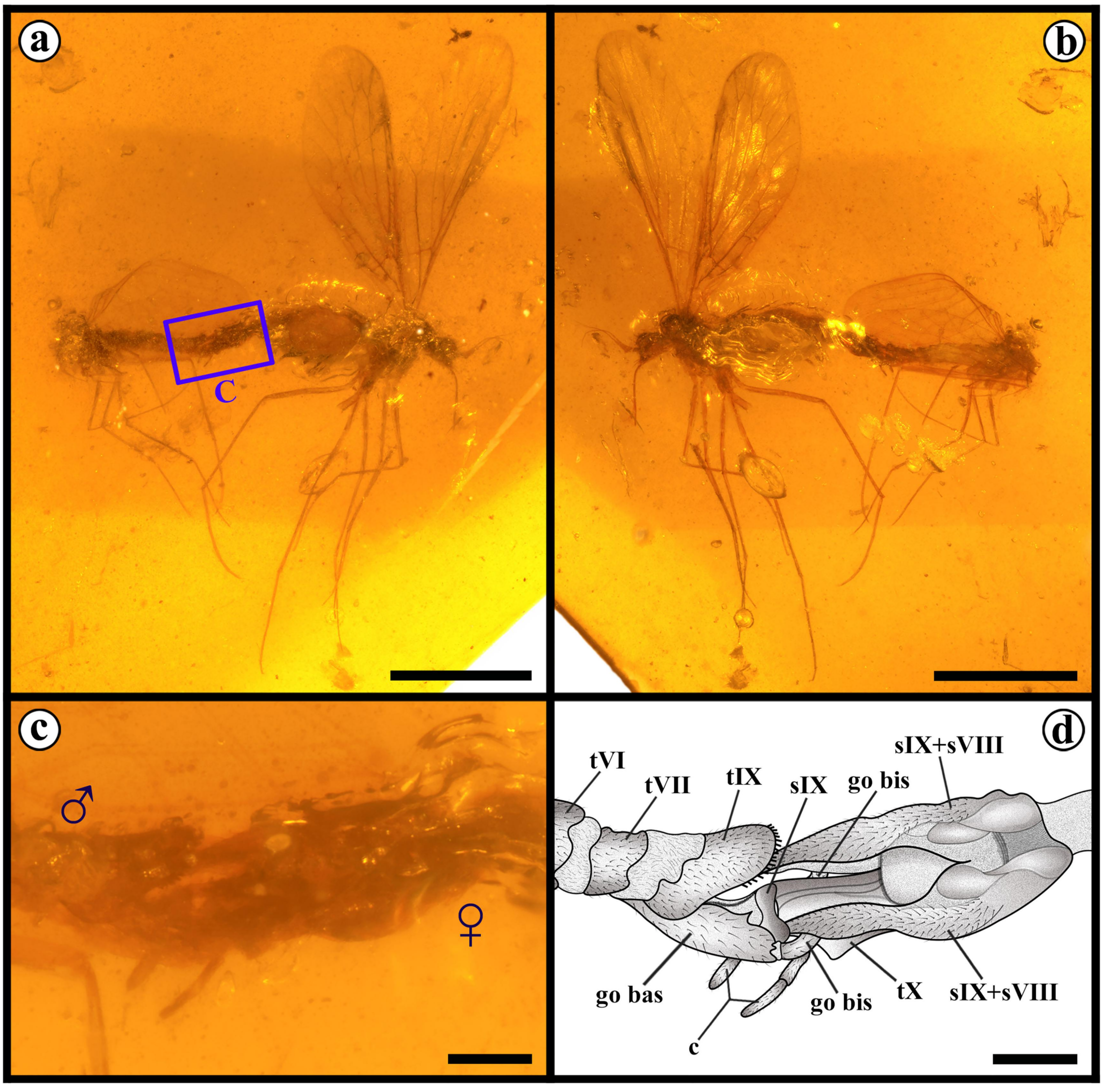

Supplementary Figure 16 | Copulating *Parapolycentropus paraburmiticus*[50]. These specimens show details of male and female genitalia (CNU-MEC-MA-2015025, new material). (**a**), Right lateral view. (**b**), Left lateral view. (**c**), Enlarged region of coupled male and female genitalia from the template in (a). (**d**), Line drawing showing details of mated terminalia, indicating inverted female genitalia. Scale bars: 2 mm in (a) and (b), and 0.2 mm in (c) and (d).

Even in amber, due to a limitation in the size of pieces, swarms of conspecific insect groups rarely are found. For *Parapolycentropus*, its body size is smaller than other known groups in Aneuretopsychina (Supplementary Data 4 and 5), and the presence of numerous individuals in an aggregation is more likely to be preserved in amber pieces of larger size. In 2014, an amber piece was reported that contained three male *Parapolycentropus* sp.[50,69], which is unlikely to record evidence of swarming behavior, as the insect number is too small for confirmation. Nevertheless, we found three additional amber pieces that consisted of abundant *Parapolycentropus* specimens with a variety of sex ratios. The first amber piece consisted of nine insects, all belonging to *Parapolycentropus paraburmiticus*[50], among them one female, six males, and two individuals of unknown sex (Supplementary Fig. 15a,b). The second amber piece was the richest one, and consisted of 18 *Parapolycentropus* sp. specimens assigned to three females, six males and nine of indeterminate sex, as well as a beetle and a small wasp, all clearly visible on both sides of the piece (Supplementary Fig. 15c,d). The third amber piece includes four scorpionflies, similar to the material in the 2014 paper[69]. This piece contains two females each of *Parapolycentropus paraburmiticus* and *Parapolycentropus burmiticus*, one male of *Parapolycentropus paraburmiticus*, one *Parapolycentropus* sp. of unknown sex, and two beetles (Supplementary Fig. 15e,f). The above amber pieces strongly suggest that these insects engaged in lekking behavior.

Additional evidence would be required to determine whether such assemblies represented a nuptial flight congregation, a mating lek or other informal gatherings. Many dipterans in mating swarms, or leks, appear to be composed mostly of males, informally termed male mating swarms, with females only periodically appearing in the lek and exiting soon after mating[130,138]. Moreover, smaller males are more likely to survive the selection process than larger ones, as they are easier to obtain one or more partners for copulation[139]. In *Parapolycentropus* and *Dualula*, males typically are smaller than females in body size but are more abundant in numbers when lekking. In the specimens mentioned above, excluding specimens of indeterminate sex, there is a definite imbalance in the male-to-female ratio. For example, the ratio of males and females in CNU-MEC-MA-2016007 is 6:1. Presently, there is evidence to indicate that some insect populations engaged in mating flight are composed overwhelmingly of males[30,138]. Nevertheless, due to an absence of sufficient fossil and modern samples, such evidence should serve as a reasonable hypothesis for further testing.

In many extant mecopterans, after the male offers a mating gift to attract the female, copulation ensues. The mating position is usually the female in the superior (upper) position, with the process controlled by the male. Simultaneously, the symmetric genitalia of the male and female are coupled, which is the primitive copulatory stance in Holometabola[139–141]. The best example of this juxtaposition of genitalia is Boreidae (snow scorpionflies), in which the male employs claspers and specialized wings to grasp the female[141,142]. However, there are some exceptions to this condition in modern mecopteran lineages, such as some Panorpidae (common scorpionflies), Panorpodidae (short-faced scorpionflies), Choristidae (Australian scorpionflies), and all Bittacidae (hangingflies). In modern *Panorpodes kuandianensis* (Panorpodidae), the male uses a pair of gonostyli to engage the female abdomen, and during copulation, the female reverses abdominal segments VII–IX by 180°[143]. There clearly are two or three phases during *P. kuandianensis* copulation. The first phase is the initial, V-shaped, side-by-side position with the abdomens converging at the V vertex. In the second phase, the shift to an end-to-end or tip-to-tip abdominal position is the final position in most individuals. This position often is modified by a third phase in some females with reversal of their body to ensure tight coupling with male genitalia[144].

The same copulation sequence exists in many Panorpidae as well[145,146]. Furthermore, in some mecopterans the male grasps the female wings or legs by notal or postnotal organs[145–147]. A few species form an O-shaped configuration in which the proboscis and genitalia of the male and female broadly connect with each other, most likely encouraged by males secreting a salivary mass as a nuptial gift to occupy the female throughout the copulation process[143]. This pattern also exists in extant Choristidae[148]. Bittacidae, however, are very different from other mecopteran groups in morphology and biology, and

importantly during copulation the male and female are face-to-face in position, and the abdomen of the male twists ca. 180° to adapt to a pendant body position during copulation[149]. In early lineages of nematocerous and lower brachycerous Diptera, excluding specialized groups, almost all genitalia are symmetrical and exhibit several copulatory positions, including male-above, false male-above, general end-to-end, and targeted tip-to-tip positions[140]. The tip-to-tip position occurs during flight is typical of lekking behaviors. Dipteran examples of this in-flight, copulatory position include Chironomidae (nonbiting midges), and some Psychodidae (moth flies), Ceratopogonidae (biting midges) and Asilidae (robber flies), wherein the male temporary reverses abdominal segments 7 and 8 by about 180° to achieve contact with genitalia of the female[150–153].

Fortunately, one piece of Myanmar Amber entombed a copulating pair of *Parapolycentropus paraburmiticus*[50]. This distinctive interaction provides the first, direct evidence for understanding the mating behavior of mid-Mesozoic Aneuretopsychina. The amber piece is dark yellow, with many small-sized impurities of detritus, and contains numerous larger air bubbles surrounding the insect bodies. The male–female scorpionfly interaction documents many microscopic structures of the genitalia as well as a distinctive copulatory position (Supplementary Fig. 16). The male is significantly smaller than the female in body size and lacks preservation of its head and proboscis (Supplementary Fig. 16a,b). (We note that female decapitation of the head or feeding on the body of the male during copulation occasionally occurs in extant nematocerous dipterans[154], but in this case, the missing male head is a taphonomic in nature.) The copulating pair presents an obvious end-to-end, or tip-to-tip, position, with segments VII and VIII, and perhaps including part of segment VI of the female abdomen that twists 180° relative to its abdomen. The male uses its claspers to grasp female sIX and sVIII but due to the highly compact integration of genitalic segments, other bound structures may be obscured (Supplementary Fig. 16c,d). Such an unconventional juxtaposition of genitalia is very rare in Mecoptera; only a few Panorpodidae, a few Bittacidae and some Nannochoristidae, a relict clade, have the same pattern of end-to-end copulation[154,155]. Among this group, only panorpodids have adopted the mode of the female abdomen rotating rather than the male abdomen rotating, as illustrated in the position of the female genitalia relative to its terminal abdominal segments (Supplementary Fig. 16c,d). A similar mode of male–female genitalia position is present in basal groups of modern Diptera[154,156–158]. Based on the above data, *Parapolycentropus* very likely engaged in a mating flight behavior, probably as part of a swarm or lek, engaged in end-to-end copulation, with female genitalia in a reversed position.

Supplementary Table 1 | Geological setting, locality, preservational status and sources for all described genera and species of Pseudopolycentropodidae.

Genus and species	Geological and locality	Preservational status	References
Parapolycentropus burmiticus	Middle Cretaceous, Kachin, Myanmar	Complete body with wings and mouthpart, male and female	[50, 69]
Parapolycentropus paraburmiticus	Middle Cretaceous, Kachin, Myanmar	Complete body with wings and mouthpart, male and female	[50, 69]
Pseudopolycentropodes virginicus	Late Triassic, Martinsville, USA	Incomplete body with wings, lack of mouthpart, sex unknown	[50]
Pseudopolycentropus daohugouensis	Middle Jurassic, Inner Mongolia, China	Incomplete body with wings and mouthpart, sex unknown	[49]
Pseudopolycentropus janeannae	Middle Jurassic, Inner Mongolia, China	Complete body with wings and mouthpart, male and female	[21,49]
Pseudopolycentropus latipennis	Late Jurassic, Karatau, Kazakhstan	Complete body with wings, female	[70, 71]
Pseudopolycentropus madygenicus	Late Jurassic, Fergana Valley, Kazakhstan	Incomplete forewing only, sex unknown	[71]
Pseudopolycentropus novokshonovi	Middle Jurassic, Inner Mongolia, China	Complete body with wings and mouthpart, female	[21,49]
Pseudopolycentropus obtusus	Upper Lias (Toarcian), Braunschweig, Germany	Incomplete forewing only, sex unknown	[72]
Pseudopolycentropus perlaeformis	Upper Lias (Toarcian), Dobbertin, Germany	Incomplete forewing only, sex unknown	[73]
Pseudopolycentropus triangularis	Upper Lias (Toarcian), Dobbertin, Germany	Complete fore- and hind wings	[74, 75]
Pseudopolycentropus triasicus	Middle Triassic (Ladinian), Arzviller, France	Incomplete forewing only, sex unknown	[76]
Sinopolycentropus rasnitsyni	Middle Jurassic, Inner Mongolia, China	Complete body with wings and mouthpart, female	[77]

Supplementary References

1. Hennig, W. *Die Stammesgeschichte der Insekten*. Vol. 49. 1–436 (Waldemar Kramer, Frankfurt am Main, 1969).
2. Boudreaux, H. B. *Arthropod Phylogeny with Special Reference to Insects*. (John Wiley & Sons, New York, 1979).
3. Kristensen, N. P. Phylogeny of endopterygote insects, the most successful lineage of living organisms. *Eur. J. Entomol.* **96,** 237–254 (1999).
4. Beutel, R. G., et al. Morphological and molecular evidence converge upon a robust phylogeny of the megadiverse Holometabola. *Cladistics* **27(4),** 341–355 (2011).
5. Pashley, D. P., McPheron, B. A. & Zimmer, E. A. Systematics of holometabolous insect orders based on 18S ribosomal RNA. *Mol. Phylo. Evol.* **2(2),** 132–142 (1983).
6. Song, N., An, S. H., Yin, X. M., Zhao, T. & Wang, X. Y. Insufficient resolving power of mitogenome data in deciphering deep phylogeny of Holometabola. *J. Syst. Evol.* **54(5),** 545–559 (2016).
7. Chalwatzis, N., Hauf, J., Van De Peer, Y., Kinzelbach, R. & Zimmerman, F. K. 18S ribosomal RNA genes of insects: Primary structure of the genes and molecular phylogeny of the Holometabola. *Ann. Entomol. Soc. Am.* **89(6),** 788–803 (1996).
8. Whiting, M. F. Mecoptera is paraphyletic: multiple genes and phylogeny of Mecoptera and Siphonaptera. *Zoologica Scripta*, **31(1),** 93–104 (2002).
9. Kristensen, N. P. Phylogeny of insect orders. *Annu. Rev. Entomol.* **26(1),** 135–157 (1981).
10. Kristensen, N. P. Forty years of insect phylogenetic systematics. *Zool. Beitr. N.F.* **36,** 83–124 (1995).
11 Wiegmann, B. M., et al. Single-copy nuclear genes resolve the phylogeny of the holometabolous insects. *BMC Biol.* **7,** 34 (2009).
12. Ishiwata, K., Sasaki, G., Ogawa, J., Miyata, T. & Su, Z. H. Phylogenetic relationships among insect orders based on three nuclear protein-coding gene sequences. *Mol. Phylo. Evol.* **58(2),** 169–180 (2011).
13. Misof, B., et al. Phylogenomics resolves the timing and pattern of insect evolution. *Science* **346(6210),** 763–767 (2014).
14. Wiegmann, B. M. et al. Single-copy nuclear genes resolve the phylogeny of the holometabolous insects. *BMC Biol.* **7,** 34 (2009).
15. Yeates, D. K., Meusemann, K., Trautwein, M., Wiegmann, B. & Zwick, A. Power, resolution and bias: recent advances in insect phylogeny driven by the genomic revolution. *Curr. Opin. Ins. Sci.* **13,** 16–23 (2016).
16. Whiting, M. F. & Wheeler, W. C. Insect homeotic transformation. *Nature* **368(6473),** 696 (1994).
17. Whiting, M. F., Carpenter, J.C., Wheeler, Q. D. & Wheeler, W. C. The Strepsiptera problem: phylogeny of the holometabolous insect orders inferred from 18S and 28S ribosomal DNA sequences and morphology. *Syst. Biol.* **46(1),** 1–68 (1997).
18. Trautwein, M. D., Wiegmann, B. M., Beutel, R., Kjer, K. M. & Yeates, D. K. Advances in insect phylogeny at the dawn of the postgenomic era. *Annu. Rev. Entom.* **57,** 449–468 (2012).
19. Peters, R. S. et al. The evolutionary history of holometabolous insects inferred from transcriptome-based phylogeny and comprehensive morphological data. *BMC Evol. Biol.* **14(1),** 52 (2014).
20. Wheeler, W. C., Whiting, M., Wheeler, Q. D. & Carpenter, J. M. The phylogeny of the extant hexapod orders. *Cladistics* **17(2),** 113–169 (2001).
21. Ren, D. et al. A probable pollination mode before angiosperms: Eurasian, long-proboscid scorpionflies. *Science* **326,** 840–847 (2009).
22. Lin, X. D., Shin, M. J. H., Labandeira, C. C. & Ren, D. New data from the Middle Jurassic of China shed light on the phylogeny and origin of the proboscis in the Mesopsychidae (Insecta: Mecoptera). *BMC Evol. Biol.* **16(1),** 1 (2016).
23. Blagoderov, V., Grimaldi, D. A. & Fraser, N. C. How time flies for flies: diverse Diptera from the Triassic of Virginia and early radiation of the order. *Am. Mus. Novit.* **3572**, 1–39 (2007).
24. Tillyard, R. J. Some new Permian insects from Belmont, NSW in the collection of Mr. John Mitchell. *Proc. Linn. Soc. N. S. W.* **47,** 279–292 (1922).
25. Tillyard, R. J. Upper Permian insects of New South Wales Part II. The orders Mecoptera, Paramecoptera and Neuroptera. *Proc. Linn. Soc. N. S. W.* **51(1),** 265–282 (1926).
26. Riek, E. F. Fossil mecopteroid insects from the Upper Permian of New South Wales. *Rec. Austral. Mus.* **23(2),** 55–87 (1953).
27. Kukalová-Peck, J. & Lawrence, J. F Relationships among coleopteran suborders and major endoneopteran lineages: evidence from hind wing characters. *Eur. J. Entomol.* **101,** 95–144 (2004).

28. Minet, J., Huang, D. Y., Wu, H. & Nel, A. Early Mecopterida and the systematic position of the Microptysmatidae (Insecta: Endopterygota). *Ann. Soc. Entomol. Fr.* **46(1-2),** 262–270 (2010).
29. Nixon, K. C. *WinClada, Version 1.00.08*. Program and Documentation. Ithaca. (Cornell University Press, New York, 2002).
30. Goloboff, P. A. *NoName (NONA), Version 2.0*. Program and Documentation. (Fundación Instituto Miguel Lillo, Tucumán, 1997).
31. Willmann, R. *Mecoptera (Insecta, Holometabola).* Fossilium Catalogus, Animalia vol. 124, 1–139 (The Hague, Junk, 1978).
32. Ponomarenko, A.G. & Rasnitsyn, A.P. Taxonomic names, in new Mesozoic and Cenozoic Protomecoptera. *Paleontol. J.* **8(4),** 493–507 (1974).
33. Martynova, O. M. New insects from Permian and Mesozoic deposits of the USSR. *Mater. Oznov. Paleontol.* **2,** 69–94 (1958) [in Russian].
34. Novokshonov, V. G. *Early Evolution of the Scorpionflies (Insecta: Panorpida).* 1-140 (Academy of Sciences, Moscow, 1997) [in Russian].
35. Handlirsch, A. *Die fossilen Insekten un die Phylogenie der rezenten Formen: ein Handbuch für Paläontologen und Zoologen*. (Wilhelm Engelmann, Leipzig, 1906–1908).
36. Grimaldi, D. A. & Engel, M. S. The relict scorpionfly family Meropeidae (Mecoptera) in Cretaceous amber. *J. Kansas Entomol. Soc*. **86(3),** 253–263 (2013).
37. Cockerell, T. D. A. Descriptions of Tertiary insects. VI. *Am. J. Sci.* (4) **27(161),** 381–387 (1909).
38. Willmann, R. Phylogenie und Verbreitungsgeschichte der Eomeropidae (Insecta: Mecoptera). Ein Beispiel für die Anwendung der phylogenetischen Systematik in der Paläontologie. *Paläontol. Z.* **55(1-2),** 31–49 (1981).
39. Qiao, X., Shih, C. K. & Ren, D. Three new species of aneuretopsychids (Insecta: Mecoptera) from the Jehol Biota, China. *Cret. Res.* **36,** 146–150 (2012).
40. Bashkuev, A. S. New Mecoptera from the end-Permian intertrappean deposits of the Tunguska Basin. *Russ. Entomol. J.* **22(1),** 1–4 (2013).
41. Tillyard, R. J. A fossil insect wing belonging to the new order Paramecoptera, ancestral to the Trichoptera and Lepidoptera from the Upper Coal-Measures of Newcastle, NSW. *Proc. Linn. Soc. N.S.W.* **44,** 231–256 (1919).
42. Tillyard, R. J. Mesozoic Insects of Queensland. No. 1: Planipennia, Trichoptera, and the new order Protomecoptera. *Proc. Linn. Soc. N. S. W.* **42,** 175–200 (1917).
43. Tillyard, R. J. Permian and Triassic insects from New South Wales, in the collection of Mr. John Mitchell. *Proc. Linn. Soc. N.S.W.* **42,** 720–756 (1918).
44. Sukatcheva, I. D. & Novokshonov, V. G. A new family of scorpionflies from the Mesozoic of Yakutia (Insecta: Mecoptera: Sibiriothaumatidae fam. nov.). *Paleontol. J.* **32,** 596–597 (1998).
45. Handlirsch, A. Neue Untersuchungen über die fossilen Insekten mit Ergänzungen und Nachträgen sowie Ausblicken auf phylogenetische, palaeogeographische und allgemein biologische Probleme. 1. Teil. *Ann. Naturhist. Mus. Wien* **48,** 1–140 (1937).
46. Martynova, O. M. 1948. Data on the evolution of the Mecoptera. *Trudy Paleontol. Inst. Akad. Nauk SSSR* **14,** 1–76 (1948).
47. Tillyard, R. J. The panorpoid complex in the British Rhaetic and Lias. *Foss. Ins. Brit. Mus. Nat. Hist*, **3,** 1–79. (1933).
48. Handlirsch, A. In *Handbuch der Entomologie* (ed. G. Schröder), vol. 3, 117–306 (G. Fischer, Jena, 1920).
49. Ren, D., Shih, C. K. & Labandeira, C. C. New Jurassic pseudopolycentropodids from China (Insecta: Mecoptera). *Acta Geol. Sin*. **84(4),** 22–30 (2010).
50. Grimaldi, D. A., Zhang, J., Fraser, N. C. & Rasnitsyn, A. P. Revision of the bizarre Mesozoic scorpionflies in the Pseudopolycentropodidae (Mecopteroidea). *Ins. Syst. Evol.* **36,** 443–458 (2005).
51. Carpenter, F. M. The affinities of *Eomerope* and *Dinopanorpa* (Mecoptera). *Psyche* **79(1-2),** 79–87 (1972).
52. Latreille, P. A. *Histoire Naturelle Générale e Particulière des Crustacés et des Insectes. Tome 2.* xii + 13–467 (F. Dufart, Paris, 1802).
53. Grimaldi, D. A. In *Studies on Fossil Amber, with Particular Reference to the Cretaceous of New Jersey* (ed. Grimaldi, D. A.) 259–303 (Backhuys, Leiden, 2000).
54. Makarkin, V. N., Yang, Q. & Ren, D. A new Cretaceous family of enigmatic two-winged lacewings (Neuroptera). *Foss. Rec.* **16(1),** 67–75 (2013).
55. Liu, X. Y., Zhang, W. W., Winterton, S. L., Breilkreuz, L. C. & Engel, M. S. Early morphological specialization for insect–spider associations in Mesozoic lacewings. *Curr. Biol.* **26(12),** 159–1594 (2016).
56. Novokshonov, V. G. & Sukatcheva, I. D. Fossil Scorpionflies of the "Suborder" Paratrichoptera (Insecta: Mecoptera). *Paleontol. Zh.* **35(2),** 173–182 (2001).

57. Ren D, Labandeira, C. C., Shih, C. K. & Rasnitsyn, A. P. New Mesozoic Mesopsychidae (Mecoptera) from northeastern China. *Acta Geol. Sin.* **84(4),** 720–731 (2010).
58. Bashkuev, A. S. The earliest Mesopsychidae and revision of the family Mesopanorpodidae (Mecoptera). *ZooKeys* **130,** 263–279 (2011).
59. Gao, T. P., Shih, C. K., Labandeira, C. C., Santiago-Blay, J. A., Yao, Y. Z. & Ren, D. Convergent evolution of ramified antennae in insect lineages from the Early Cretaceous of Northeastern China. *Proc. R. Soc. B* **283,** 20161448 (2016).
60. Lewis, E. B. A gene complex controlling segmentation in *Drosophila*. *Nature* **276,** 565–570 (1978).
61. Akam, M., Averof, M., Castelli-Gair, J., Dawes, R., Falciani, F. & Ferrier, D. The evolving role of Hox genes in arthropods. *Development* **1994(Supplementary),** 209–215 (1994).
62. Ronshaugen, M., Biemar, F., Piel, J., Levine, M. & Lai, E. The Drosophila microRNA lab-4 causes a dominant homeotic transformation of halteres to wings. *Genes Devel.* **19(24),** 2947–2952 (2005).
63. Mohit, P., Bajpai, R. & Shashidhara, L. S. Regulation of wingless and vestigial expression in wing and haltere discs of *Drosophila*. *Development* **130(8),** 1537–1547 (2003).
64. Mohit, P., et al. Modulation of AP and DV signaling pathways by the homeotic gene *Ultrabithorax* during haltere development in *Drosophila*. *Devel. Biol.* **291(2),** 356–367 (2006).
65. Pallavi, S. K., Kannan, R. & Shashidhara, L. S. Negative regulation of Egfr/Ras pathway by *Ultrabithorax* during haltere development in *Drosophila*. *Devel. Biol.* **296(2),** 340–352 (2006).
66. Crickmore, M. A. & Mann, R. S. Hox control of organ size by regulation of morphogen production and mobility. *Science* **313(5783),** 63–68 (2006).
67. Makhijani, K., Kalyani, C., Srividya, T. & Shashidhara, L. S. Modulation of decaptentaplegic gradient during haltere specification in *Drosophila*. *Devel. Biol.* **302(1),** 243–255 (2007).
68. Navas, de L. F., Garaulet, D. L. & Sánchez-Herrero, E. The *Ultrabithorax* Hox gene of *Drosophila* controls haltere size by regulation of the DPP pathway. *Development* **133(22),** 4495–4506 (2006).
69. Grimaldi, D. A. & Johnston, M. A. The long-tongued Cretaceous scorpionfly *Parapolycentropus* Grimaldi and Rasnitsyn (Mecoptera: Pseudopolycentropodidae): new data and interpretations. *Am. Mus. Novit.* **3793,** 1–24 (2014).
70. Martynov, A. V. To the knowledge of fossil insects from Jurassic beds in Turkestan. 3. Hymenoptera, Mecoptera. *Izv. Ross. Akad. Nauk*, *Ser. 6*, **19(16-17),** 753–762 (1925).
71. Novokshonov, V. G. New Triassic scorpionflies (Insecta, Mecoptera). *Paleontol. J.* **31,** 628–635 (1997).
72. Bode, A. Die Insektenfauna des ostneidersächsischen Oberen Lias. *Palaeontographica* **103,** 1–375 (1953).
73. Geinitz, F. E. Die Flötzformationen Mecklenburgs. *Archiv Ver. Freu. Naturges. Meck.* **37,** 7–151 (1884).
74. Handlirsch, A. in *Handbuch der Entomologie. Band III: Geschichte, Literatur, Technik, Paläontologie, Phylogenie, Systematik* (ed Schröder) 307–376 (Fischer, Jena, 1925).
75. Ansorge. J. Insekten aus dem oberen Lias von Grimmen (Vorpommern, Norddeutschland). *Neue Paläont. Abh.* **2,** 1–132 (1996).
76. Papier, F., Nel, A. & Grauvogel-Stamm L. Deux nouveaux insectes Mecopteroidea du Buntsandstein supérieur (Trias) des Vosges. *Paleont. Lomb.* **5,** 37–45 (1996).
77. Shih, C. K., Yang, X. G., Labandeira, C. C. & Ren, D. A new long-proboscid genus of Pseudopolycentropodidae (Mecoptera) from the Middle Jurassic of China and its plant-host specializations. *ZooKeys* **130,** 281–297 (2011).
78. Lambkin, K. J. The Mesopsychidae (Mecoptera) of the Queensland Triassic. *Austral. Entomol.* **41,** 135–146 (2014).
79. Bashkuev, A. S. Nedubroviidae, a new family of Mecoptera: the first Paleozoic long-proboscid scorpionflies. *Zootaxa* **2895,** 47–57 (2011).
80. Novokshonov, V. G., Sukacheva, I. D. & Aristov, D. S. An Early Triassic scorpionfly (Panorpida = Mecoptera) of the Vologda Region. *Paleontol. J.* **38(Supplementary 2),** S214–S215 (2004).
81. Willmann, R., Diener, C., Frech, F., Pompeckj, J. F., Quenstedt, W. & Westphal, F. *Mecoptera (Insecta, Holometabola).* Fossilium Catalogus, Animalia. vol. 124, 1–134 (The Hague, Junk, 1978).
82. Krzemiński, W. & Krzemińska, E. Triassic Diptera: descriptions, revisions and phylogenetic relations. *Acta Zool. Cracov.* **46(Supplementary),** 153–184 (2003).
83. Ren, D., Shih, C. K. &Labandeira, C. C. A well-preserved aneuretopsychid from the Jehol Biota of China (Insecta, Mecoptera, Aneuretopsychidae). *ZooKeys* **129,** 17–28 (2011).
84. Labandeira, C. C. The pollination of mid Mesozoic seed plants and the early history of long-proboscid insects. *Ann. Missouri Bot. Gard.* **97,** 469–513 (2010).
85. Rasnitsyn, A. P. & Kozlov, M. V. A new group of fossil insects: a scorpionfly with adaptations of cicads and moths. *Doklady Akad. Nauk SSSR* **310(4),** 973–976 (1990).

86. Labandeira, C. C. et al. The evolutionary convergence of mid-Mesozoic lacewings and Cenozoic butterflies. *Proc. R. Soc. B* **283,** 20152893 (2016).
87. Shih, C. K., Qiao, X., Labandeira, C. C. & Ren, D. A new mesopsychid (Mecoptera) from the Middle Jurassic of Northeastern China. *Acta Geol. Sin.-Engl. Edn.* **87,** 1235–1241 (2013).
88. Arillo, A., et al. Long-proboscid brachyceran flies in Cretaceous amber (Diptera: Stratiomyomorpha: Zhangsolvidae). *Syst. Entomol*. **40,** 242–267 (2015).
89. Peñalver, E., et al. Long-proboscid flies as pollinators of Cretaceous gymnosperms. *Curr. Biol.* **25,** 1917–1923 (2015).
90. Makarkin, V. N. Enormously long, siphonate mouthparts of a new, oldest known spongillafly (Neuroptera, Sisyridae) from Burmese amber imply nectarivory or hematophagy. *Cret. Res.* **65,** 126–137 (2016).
91. Chaudonneret, J. *Les Pièces Buccales des Insectes: Thème et Variations*. 1–256 (Edition hors Serie du Bulletin Scientifique de Bourgogne, Dijon, 1990).
92. Eastham, L. E. S. & Eassa, Y. E. E. The feeding mechanism of the butterfly, *Pieris brassicae* L. *Philos. Trans. R. Soc. Lond. B* **239,** 1–43 (1955).
93. Kingsolver, J. G. & Daniel, T. L. On the mechanics and energetics of nectar feeding in butterflies. *J. Theor. Biol.* **76,** 167–169 (1979).
94. Labandeira, C. C., Kvaček, J. & Mostovski, M. B. Pollination drops, pollen, and insect pollination of Mesozoic gymnosperms. *Taxon* **56,** 663–695 (2007).
95. McLean, D. L. & Kinsey, M. G. The precibarial valve and its role in the feeding behavior of the pea aphid, *Acyrthosiphon pisum*. Bull. Entomol. Soc. Am. **30,** 26–31 (1984).
96. Labandeira, C. C. in *The Evolutionary Biology of Flies* (eds. Yeates D. K. & Wiegmann B. M.) 217–273. (Columbia, Univ. Press, New York, 2005).
97. Peñalver, E. et. al. Thrips pollination of Mesozoic gymnosperms. *Proc. Natl. Acad. Sci. USA* **109,** 8623–8628 (2012).
98. Peris, D, Pérez-de la Fuente, Peñalver, E., Delclòs, X., Barrón, E. & Labandeira, C. C. False blister beetles and the expansion of gymnosperm-insect pollination modes before angiosperm dominance. *Curr. Biol.* **27,** 897–904 (2017).
99. Cai, C., Escalona, H. E., Li, L., Yin, Z., Huang, D. & Engel, M. S. Beetle pollination of cycads in the Mesozoic. *Curr. Biol*. **28,** 1–7 (2018).
100. Friis, E. M., Pederson, K. R. & Crane, P. R. *Early Flowers and Angiosperm Evolution*. (Columbia Univ. Press, New York, 2011).
101. Zetter, R., Hesse, M. & Huber, K. H. Combined LM, SEM and TEM studies of Late Cretaceous pollen and spores from Gmiind, Lower Austria. *Stapfia*, **80,** 201–230 (2002).
102. Crepet, W. L., Friis, E. M. & Nixon, K. C. Fossil evidence for the evolution of biotic pollination. *Philos. Trans. R. Soc. Lond. B* **333,** 187–195 (1991).
103. Solórzano Kraemer, M. M., Perrichot, V., Brown, B. V., Tafforeau, P. & Soriano, C. A new species of the Cretaceous genus *Prioriphora* (Diptera: Phoridae) in French amber. *Syst. Entom.* **36,** 581–588 (2011).
104. Labandeira, C. C. In *Evolutionary Biology, Genomes, Evolution, Speciation, Coevolution and Origin of Life* (ed. P. Pontarotti) 261–299 (Springer International Publishing, Switzerland, 2014).
105. Anderson, J. M. & Anderson, H. M. Heyday of the gymnosperms: Systematics and biodiversity of the Late Triassic Molteno fructifications. *Strelitzia* **15,** 1–138 (2003).
106. Chambers, K. L., Poinar, G. Jr. & Buckley, R. *Tropidogyne*, a new genus of Early Cretaceous Eudicots (Angiospermae) from Burmese amber. *Novon* **20,** 23–29 (2010).
107. Poinar, G. O. Jr. & Chambers, K. L. *Tropidogyne pentaptera* sp. nov., a new mid-Cretaceous fossil angiosperm flower in Burmese amber. *Palaeodiversity* **10,** 135–140 (2017).
108. Thien, L. B., Azuma, H. & Kawano, S. New perspectives on the pollination biology of basal angiosperms. *Int. J. Plant Sci.* **161,** S225–S235 (2000).
109. Jervis, M. Functional and evolutionary aspects of mouthpart structure in parasitoid wasps. *Biol. J. Linn. Soc*. **63,** 461–493 (1998).
110. Sun, K. Q., Cui, J. Z. & Wang, S. J. *Volume 3: Fossil Gymnosperms in China (I).* (ed. Cui, J.) 1–383, 224 pls. (Higher Education Press, Beijing, 2016)
111. Wang, S.-J., Cui, J.-Z., Yang, Y. & Sun, K. Q. *Volume 3: Fossil Gymnosperms in China (II).* (ed. Cui, J. S.) 1–417, 280 pls. (Higher Education Press, Beijing, 2016)
112. Huang, D. Y. et al. *The Daohugou Biota*. (Shanghai Science and Technology Press, Shanghai, 2016).
113. Powell, J. A. & Opler, P. A. *Moths of Western North America*. 1–536 (University of California Press, Berkeley, 2009).

114. Carroll, S. B., Weatherbee, S. D. & Langeland, J. A. Homeotic genes and the regulation and evolution of insect wing number. *Nature* **375(6526),** 58–61 (1995).
115. Weatherbee, S. D., Halder, G., Kim, J., Hudson, A. & Carroll, S. *Ultrabithorax* regulates genes at several levels of the wing-patterning hierarchy to shape the development of the *Drosophila* haltere. *Genes Develop*. **12(10),** 1474–1482 (1998).
116. Clark-Hatchel, C. M. & Tomoyasu, Y. (2016) Exploring the origin of insect wings from an evo-devo perspective. *Curr. Opin. Ins. Sci.* **13,** 77–85 (2016).
117. González-Gaitán, M. A., Micol, J. L. & García-Bellido, A. Developmental genetic analysis of *Contrabithorax* mutations in *Drosophila melanogaster*. *Genetics* **126(1),** 139–155 (1990).
118. Pavlopoulos, A. & Akam, M. Hox gene *Ultrabithorax* regulates distinct sets of target genes at successive stages of *Drosophila* haltere morphogenesis. *Proc. Natl. Acad. Sci. U. S. A.* **108(7),** 2855–2860 (2011).
119. Pringle, J. W. S. The gyroscopic mechanism of the halteres of Diptera. *Phil. Trans. R. Soc. Lond*. **233(602),** 347–384 (1948).
120. Bender, J. A. & Dickinson, M. H. A comparison of visual and haltere-mediated feedback in the control of body saccades in *Drosophila melanogaster*. *J. Expt. Biol.* **209(23),** 4597–4606 (2006).
121. Deora, T., Singh, A. K. & Sane, S. P. Biomechanical basis of wing and haltere coordination in flies. *Proc. Natl. Acad. Sci.* **112(5),** 1481–1486 (2015).
122. Sherman, A. & Dickinson, M. H. A comparison of visual and haltere-mediated equilibrium reflexes in the fruit fly *Drosophila melanogaster*. *J. Expt. Biol.* **206(2),** 295–302 (2003).
123. Fox, J. L. & Daniel, T. L. A neural basis for gyroscopic force measurement in the halteres of *Holorusia*. *J. Comp. Physiol. A* **194(10),** 887–897 (2008).
124. Grimaldi, D. & Engel, M. S. *Evolution of the Insects*. (Cambridge Univ. Press, New York, 2005).
125. Carpenter F. M. In *Treatise on Invertebrate Paleontology: Part 4 Arthropoda, Vol. 3 Superclass Hexapoda* (73–75, 361–362). (Geological Society of America, Boulder, Colorado, 1992).
126. Krzemiński, W. & Krzemińska, E. Revision of *Laurentiptera gallica* from the Lower/Middle Triassic of France (Mecoptera: Liassophilidae). *Pol. Pismo Entomol.* **65(5),** 267–274 (1996).
127. Novokshonov, V. G. Scorpionflies of the Family Liassophilidae from the Triassic of Ukraine. *Paleontol. J.* **36(4),** 380–382 (2002).
128. Walker, J. D., Geissman, J. W., Bowring, S. A. & Babcock, L. E. The Geological Society of America geologic time scale. *Geol. Soc. Am. Bull.* **125(3-4),** 259–272 (2013).
129. He, H. Y., et al. ^{40}Ar/^{39}Ar dating of ignimbrite from Inner Mongolia, northeastern China indicates a post-middle Jurassic age for the Daohugou Bed. *Geophys Res. Lett.* **31(20),** 206–209 (2004).
130. Downes, J. A. The swarming and mating flight of Diptera. *Annu. Rev. Entomol.* **14(1),** 271–298 (1969).
131. Martínez-Delclòs, X, Briggs D. E. G. & Peñalver, E. Taphonomy of insects in carbonates and amber. *Palaeogeogr Palaeoclimat Palaeoecol* **203,** 19–64 (2004).
132. Arillo, A. Paleoethology: fossilized behaviours in amber. *Geol. Acta* **5(2),** 159–166 (2007).
133. Barden, P. & Grimaldi, D. A. Adaptive radiation in socially advanced stem-group ants from the Cretaceous. *Curr. Biol.* **26(4),** 515–521 (2016).
134. Peñalver, E. & Grimaldi, D. A. Assemblages of mammalian hair and blood-feeding midges (Insecta: Diptera: Psychodidae: Phlebotominae) in Miocene amber. *Trans. R. Soc. Edinburgh* **96(2),** 177–195 (2005).
135. Grimaldi, D. A, Cumming, J. M. & Arillo, A. Chimeromyiidae, a new family of Eremoneuran Diptera from the Cretaceous. *Zootaxa* **2078,** 34–54 (2009).
136. Perkovsky, E. E. & Rasnitsyn, A. P. Biting midges (Diptera, Ceratopogonidae) in amber forest communities based on analysis of syninclusions in Late Eocene Rovno amber. *Terr. Arth. Rev.* **6(1-2),** 71–80 (2013).
137. Wang, M. Q., Chen, H. Y. & Yang, D. Swarming of Diptera insects. *Chin. Bull. Entom.* **47(6),** 1280–1286 (2010) [in Chinese].
138. Thornhill, R. Sexual selection within mating swarms of the lovebug, *Plecia nearctica* (Diptera: Bibionidae). *Anim. Behav.* **28,** 405–412 (1980).
139. McLachlan, A. J. & Allen, D. F. Male mating success in Diptera: advantages of small size. *Oikos* **48(1),** 11–14 (1987).
140. Huber, B. A., Sinclair, B. J. & Schmitt, M. The evolution of asymmetric genitalia in spiders and insects. *Biol. Rev.* **82(4),** 647–698 (2007).
141. Cooper, K. W. Sexual biology, chromosomes, development, life histories and parasites of *Boreus*, especially of *B. notoperates*, A southern California *Boreus*. II. (Mecoptera: Boreidae). *Psyche* **81(1),** 84–120 (1974).
142. Mickoleit, G. & Mickoleit, E. Über die funktionelle Bedeutung der Tergalapophysen von *Boreus westwoodi* (Hagen) (Insecta, Mecoptera). *Zoomorphologie* **85(2),** 157–164 (1976).

143. Zhong, W., Zhang, J.X. & Hua, B.Z. *Panorpodes kuandianensis*, a new species of short-faced scorpionflies (Mecoptera, Panorpodidae) from Liaoning, China. *Zootaxa*, **2921(1),** 47–55 (2011).
144. Tong, X., Jiang, L. & Hua, B. Z. A unique mating pattern of *Panorpodes kuandianensis* (Mecoptera: Panorpodidae). *Contrib. Zool.* **86(3),** 229–237 (2017).
145. Ma, N. & Hua, B. Z. Structural evidence why males of *Panorpa liui* offer prey rather than salivary mass as their nuptial gift. *Acta Zool.* **92(4),** 398–403 (2011).
146. Zhong, W. & Hua, B. Mating Behaviour and copulatory mechanism in the scorpionfly *Neopanorpa longiprocessa* (Mecoptera: Panorpidae). *PLoS One* **8(9),** e74781 (2013).
147. Ma, N., Zhong, W & Hua, B. Z. Genitalic morphology and copulatory mechanism of the scorpionfly *Panorpa jilinensis* (Mecoptera: Panorpidae). *Micron* **41(8),** 931–938 (2010).
148. Byers, G. W. & Thornhill, R. Biology of the Mecoptera. *Annu. Rev. Entomol.* **28(1),** 203–228 (1983).
149. Gua, Q. H. & Hua, B. Z. Co-evolution of the mating position and male genitalia in insects: a case study of a hangingfly. *PLoS ONE* **8(12),** e80651 (2013).
150. Syrjämäki, J. Swarming and mating behaviour of *Allochironomus crassiforceps* Kieff. (Dipt., Chironomidae). *Ann. Zool. Fenn.* **1(2),** 125–145 (1964).
151. Jarvis, E. K. & Rutledge, L. C. Laboratory observations on mating and leklike aggregations in *Lutzomyia longipalpis* (Diptera: Psychodidae). *J. Med. Entomol.* **29(2),** 171–177 (1992).
152. Tokeshi, M. & Reinhardt, K. Reproductive behaviour in *Chironomus anthracinus* (Diptera: Chironomidae), with a consideration of the evolution of swarming. *J. Zool.* **240(1),** 103–112 (1996).
153. Lavigne, R. J. Evolution of courtship behaviour among the Asilidae (Diptera), with a review of courtship and mating. *Stud. Dipterol.* **9,** 703–742 (2002).
154. Downes, J. A. Feeding and mating in the insectivorous Ceratopogoninae (Diptera). *Mem. Entomol. Soc. Canada* **110(S104),** 1–62 (1978).
155. Bornemissza, G. F. Observations on the hunting and mating behaviour of two species of scorpion flies (Bittacidae: Mecoptera). *Austral. J. Zool.* **14(3),** 371–382 (1966).
156. Kaltenbach, A. *Mecoptera (Schnabelkerfe, Schnabelfliegen)*. Handbuch der Zoologie, IV. Arthropoda, 2. Insecta **28,** 1–111 (1978).
157. Yeates, D. K. The cladistics and classification of the Bombyliidae (Diptera: Asiloidea). *Bull. Am. Mus. Nat. Hist.* **219,** 1–191 (1994).
158. Clements, A. N. *The Biology of Mosquitoes. Volume 2: Sensory Reception and Behaviour.* 1–740 (CABI Publishing, Wallingford, England, 1999).
159. Shi, G. H., et al. Age constraint on Burmese amber based on U-Pb dating of zircons. *Cret. Res.* **37,** 155–163 (2012).
160. Buffington, M. L., Burks, R. A. & McNeil, L. Advanced techniques for imaging parasitic Hymenoptera (Insecta). *Am. Entomol.* **51(1),** 50–56 (2005).
161. Kerr, P. H., Fisher, E. M. & Buffington, M. L. Dome lighting for insect imaging under a microscope. *Am. Entomol.* **54(4),** 198–200 (2008).
162. Buffington, M. & Gates, M. Advanced imaging techniques II: using a compound microscope for photographing point-mount specimens. *Am. Entomol.* **54(4),** 222–224 (2008).
