# Supplementary material for: Life habits and evolutionary biology of new two-winged long-proboscid scorpionflies from mid-Cretaceous Myanmar amber
Source: Nat Commun. 2019 Mar 15;10:1235. doi: 10.1038/s41467-019-09236-4 (PMC6420582; doi:10.1038/s41467-019-09236-4)
Supplement: Supplementary file 3 — Description of Additional Supplementary Files [file 41467_2019_9236_MOESM3_ESM.pdf]

Description of Additional Supplementary Files

File Name: Supplementary Data 1
Description: The 51 morphological characters and their states.

File Name: Supplementary Data 2
Description: Character-state matrix of 51 characters coding the 37 taxa in the phylogenetic analysis.

File Name: Supplementary Data 3
Description: Length x width measurements of Cycadopites sp. pollen grains at or adjacent to the body surface of specimen CNU-MEC-MA2017012.

File Name: Supplementary Data 4
Description: Head, proboscis, antenna and body measurements and their aspect-ratio values of amber Dualulidae and compression Aneuretopsychina.

File Name: Supplementary Data 5
Description: Head, proboscis, antennal body measurements and their aspect-ratio values for Parapolycentropus from new Myanmar amber material.

File Name: Supplementary Data 6
Description: Various corolla measurements of flower specimens from Myanmar amber.

File Name: Supplementary Movie 1
Description: Micro-CT scanning of head and mouthparts of Parapolycentropus paraburmiticus. CNU-MEC-MA-2017008, new material, male. This video displays serial sections of the proboscis base in left lateral and dorsal views, attached to Supplementary Figure 9."
