# Supplementary material for: Life habits and evolutionary biology of new two-winged long-proboscid scorpionflies from mid-Cretaceous Myanmar amber
Source: Nat Commun. 2019 Mar 15;10:1235. doi: 10.1038/s41467-019-09236-4 (PMC6420582; doi:10.1038/s41467-019-09236-4)
Supplement: Supplementary file 11 — Reporting Summary [file 41467_2019_9236_MOESM11_ESM.pdf]

natureresearch

Corresponding author(s): Dong Ren

Last updated by author(s): Feb 27, 2019

Reporting Summary

Nature Research wishes to improve the reproducibility of the work that we publish. This form provides structure for consistency and transparency in reporting. For further information on Nature Research policies, see Authors & Referees and the Editorial Policy Checklist.

Statistics

For all statistical analyses, confirm that the following items are present in the figure legend, table legend, main text, or Methods section.

n/a	Confirmed	
☐	☒	The exact sample size (n) for each experimental group/condition, given as a discrete number and unit of measurement
☐	☒	A statement on whether measurements were taken from distinct samples or whether the same sample was measured repeatedly
☒	☐	The statistical test(s) used AND whether they are one- or two-sided<br>*Only common tests should be described solely by name; describe more complex techniques in the Methods section.*
☒	☐	A description of all covariates tested
☒	☐	A description of any assumptions or corrections, such as tests of normality and adjustment for multiple comparisons
☐	☒	A full description of the statistical parameters including central tendency (e.g. means) or other basic estimates (e.g. regression coefficient) AND variation (e.g. standard deviation) or associated estimates of uncertainty (e.g. confidence intervals)
☒	☐	For null hypothesis testing, the test statistic (e.g. F, t, r) with confidence intervals, effect sizes, degrees of freedom and P value noted<br>*Give P values as exact values whenever suitable.*
☒	☐	For Bayesian analysis, information on the choice of priors and Markov chain Monte Carlo settings
☒	☐	For hierarchical and complex designs, identification of the appropriate level for tests and full reporting of outcomes
☒	☐	Estimates of effect sizes (e.g. Cohen's d, Pearson's r), indicating how they were calculated

Our web collection on statistics for biologists contains articles on many of the points above.

Software and code

Policy information about availability of computer code

Data collection	No other special software used during the building of character-state matrix.
Data analysis	The character-state matrix was entered into WinClada (Version1.00.08) and parsimony analyses were performed by NONA (Version 2.0).

For manuscripts utilizing custom algorithms or software that are central to the research but not yet described in published literature, software must be made available to editors/reviewers. We strongly encourage code deposition in a community repository (e.g. GitHub). See the Nature Research guidelines for submitting code & software for further information.

Data

Policy information about availability of data

All manuscripts must include a data availability statement. This statement should provide the following information, where applicable:

- Accession codes, unique identifiers, or web links for publicly available datasets
- A list of figures that have associated raw data
- A description of any restrictions on data availability

The authors declare that the data supporting the findings of this study are available within the paper and its supplementary information files. Higher-resolution versions of the figures (https://doi.org/10.6084/m9.figshare.7775801.v1) and supplementary data (https://doi.org/10.6084/m9.figshare.7775822.v1) have been deposited in the figshare database. All relevant data are available from the corresponding authors upon request.

Field-specific reporting

Please select the one below that is the best fit for your research. If you are not sure, read the appropriate sections before making your selection.

☒ Life sciences ☐ Behavioural & social sciences ☐ Ecological, evolutionary & environmental sciences

For a reference copy of the document with all sections, see nature.com/documents/nr-reporting-summary-flat.pdf

Life sciences study design

All studies must disclose on these points even when the disclosure is negative.

Sample size	The authors declare that the data supporting the findings of this study are available within the paper and its supplementary information files. Higher-resolution versions of the figures (https://doi.org/10.6084/m9.figshare.7775801.v1) and supplementary data (https://doi.org/10.6084/m9.figshare.7775822.v1) have been deposited in the figshare database. All relevant data are available from the corresponding authors upon request.
Data exclusions	No data excluded from the analysis.
Replication	We make sure this study can be repeated under the same conditions.
Randomization	In the phylogenetic analysis, sample selection is not random. Type genera were used for most families in the analyses. However, if the type genera for some families lacked a full or nearly full complement of features, we employed non-type genera that were more complete morphologically to represent the full or fullest character set achievable for the given taxon. The selection of outgroups was based on the phylogenetic results of the Misof et al., 2014 analysis13 that showed insect relationships among dipterans, mecopterans and related taxa, including stem-groups.
Blinding	In the phylogenetic analysis, sample selection is not random. Type genera were used for most families in the analyses. However, if the type genera for some families lacked a full or nearly full complement of features, we employed non-type genera that were more complete morphologically to represent the full or fullest character set achievable for the given taxon. The selection of outgroups was based on the phylogenetic results of the Misof et al., 2014 analysis13 that showed insect relationships among dipterans, mecopterans and related taxa, including stem-groups.

Reporting for specific materials, systems and methods

We require information from authors about some types of materials, experimental systems and methods used in many studies. Here, indicate whether each material, system or method listed is relevant to your study. If you are not sure if a list item applies to your research, read the appropriate section before selecting a response.

Materials & experimental systems

n/a	Involved in the study
☒	☐ Antibodies
☒	☐ Eukaryotic cell lines
☐	☒ Palaeontology
☒	☐ Animals and other organisms
☒	☐ Human research participants
☒	☐ Clinical data

Methods

n/a	Involved in the study
☒	☐ ChIP-seq
☒	☐ Flow cytometry
☒	☐ MRI-based neuroimaging

Palaeontology

Specimen provenance	We confirm that all fossil samples are not from protected sites and not in conflict with the local government policies.
Specimen deposition	Most of the studied material is housed in the Key Lab of Insect Evolution and Environmental Changes, at the College of Life Sciences of Capital Normal University (CNU), in Beijing, China. Six specimens of CNU-MEC-MA-2015025, CNU-MEC-MA-2015027, CNU-MEC-MA-2015029, CNU-MEC-MA-2015030, CNU-MEC-MA-2015031 and CNU-MEC-MA-2015032 currently are on loan to CNU but will be returned to the Three Gorges Entomological Museum (EMTG), in Chongqing, China, where they finally will reside.
Dating methods	No new dates provided.

☒ Tick this box to confirm that the raw and calibrated dates are available in the paper or in Supplementary Information.
